# Overexpression of *Trypanosoma cruzi* High Mobility Group B protein (*Tc*HMGB) alters the nuclear structure, impairs cytokinesis and reduces the parasite infectivity

**DOI:** 10.1038/s41598-018-36718-0

**Published:** 2019-01-17

**Authors:** Luis Emilio Tavernelli, Maria Cristina M. Motta, Camila Silva Gonçalves, Marcelo Santos da Silva, Maria Carolina Elias, Victoria Lucia Alonso, Esteban Serra, Pamela Cribb

**Affiliations:** 1 Instituto de Biología Molecular y Celular de Rosario (IBR), Consejo Nacional de Investigaciones Científicas y Técnicas (CONICET), Rosario, Argentina; 2 Universidad Nacional de Rosario (UNR), Facultad de Ciencias Bioquímicas y Farmacéuticas, Cátedra de Parasitología, Rosario, Argentina; 3 Instituto de Biofísica Carlos Chagas Filho, Universidade Federal do Rio de Janeiro, Laboratorio de Ultraestrutura Celular Hertha Meyer, Rio de Janeiro, Brazil; 4 Laboratório Especial de Ciclo Celular, Instituto Butantan, São Paulo, SP 05503-900 Brazil; 5 Center of Toxins, Immune Response and Cell Signaling – CeTICS, Instituto Butantan, São Paulo, SP 05503-900 Brazil

## Abstract

Kinetoplastid parasites, included *Trypanosoma cruzi*, the causal agent of Chagas disease, present a unique genome organization and gene expression. Although they control gene expression mainly post-transcriptionally, chromatin accessibility plays a fundamental role in transcription initiation control. We have previously shown that High Mobility Group B protein from *Trypanosoma cruzi* (*Tc*HMGB) can bind DNA *in vitro*. Here, we show that *Tc*HMGB also acts as an architectural protein *in vivo*, since the overexpression of this protein induces changes in the nuclear structure, mainly the reduction of the nucleolus and a decrease in the heterochromatin:euchromatin ratio. Epimastigote replication rate was markedly reduced presumably due to a delayed cell cycle progression with accumulation of parasites in G2/M phase and impaired cytokinesis. Some functions involved in pathogenesis were also altered in *Tc*HMGB-overexpressing parasites, like the decreased efficiency of trypomastigotes to infect cells *in vitro*, the reduction of intracellular amastigotes replication and the number of released trypomastigotes. Taken together, our results suggest that the *Tc*HMGB protein is a pleiotropic player that controls cell phenotype and it is involved in key cellular processes.

## Introduction

*Trypanosoma cruzi* is a hemoflagellate protozoan parasite and it is the causative agent of Chagas disease, a neglected disease endemic in Latin America. There are currently limited options for safe pharmacological treatment: the only available drugs for treatment, Benznidazole and Nifurtimox, have proven to be efficient during the acute phase while its use during the chronic phase is still controversial^1^. *T*. *cruzi* has a complex life cycle that alternates between replicative and non-replicative forms in insect and mammalian hosts. Intracellular amastigotes and bloodstream trypomastigotes are present in the mammalian host, whereas epimastigotes and the infective metacyclic trypomastigotes are present in insect vectors from the Reduviidae family^2^. Through its life cycle, the trypanosome’s nuclear structure undergoes several changes. The epimastigote form, which divides by binary fission in the triatomine insect gut, presents a rounded nucleus with a defined nucleolus and relatively small amounts of peripheral heterochromatin. A similar pattern is found in the intracellular amastigotes nuclei. On the other hand, the non-replicative trypomastigote forms, exhibit an elongated nucleus, no identifiable nucleolus and heterochromatin distributed quite homogeneously through the whole nucleoplasm. These changes are accompanied by a decrease in transcription rates when the replicative forms transform into trypomastigote forms^3,4^. It is not fully understood, however, how these differences in the nuclear structure are achieved during the differentiation process.

High Mobility Group B (HMGB) proteins are highly abundant ubiquitous non-histone chromatin proteins. They play fundamental roles both inside the nucleus, where they act as architectural factors and outside the cell, where they function as alarmins participating in cell signaling and inflammation^5–7^. These proteins possess one or two HMG-box domains capable of recognizing and binding altered DNA structures with high affinity. Upon binding, HMGBs bend the DNA helix thus being able to alter the chromatin structure. Thus, HMGBs are considered architectural factors and they are involved in key nuclear processes like transcriptional control, DNA replication, recombination and repair^8,9^. Mammalian HMGB1, as well as most higher eukaryotic HMGBs, bear two “HMG-box” domains in tandem named “A-box” and “B-box” followed by about 30 glutamic and aspartic amino acids known as the “C-terminal acidic tail”, which modulates the DNA-binding properties and functioning of these proteins^10^. Kinetoplastid parasites, including the *Trypanosoma cruzi*, also encode ortholog proteins from the HMGB family with two HMG box domains, in contrast to other HMGBs identified so-far in protozoan parasites like *Plasmodium falciparum*, *Toxoplasma gondii*, and *Entamoeba histolytica* that bear only one HMG-box^11–14^. The HMGBs from kinetoplastid protozoa lack the typical acidic tail in the C-terminus, and have, instead, a unique sequence of 110 amino acids in the N-terminus conserved among trypanosomatid HMGBs and absent in all other HMGB family members. According to Pfam (http://pfam.sanger.ac.uk/) and SUPERFAMILY (http://supfam.cs.bris.ac.uk/SUPERFAMILY/), the trypanosomatid HMGBs contain a “DEK-C terminal domain”, defined as a DNA binding structural domain found in the C-terminal region of the chromatin-associated oncoprotein DEK^15^. This N-terminal region also bears a predicted Nuclear Localization Signal (NLS), which differs, in sequence and in location, from human HMGB1’s NLSs^16^.

In our previous work, we demonstrated that *Tc*HMGB is a nuclear protein expressed in all *T*. *cruzi* life cycle stages. Interestingly, replicative forms of the parasite showed higher levels of *Tc*HMGB than the infective non replicative trypomastigote forms, what may be related to the chromatin structure changes in the parasite^16^. Using a recombinant *Tc*HMGB protein, we showed that the *T*. *cruzi* HMGB, has architectural features like the ability to bend linear DNA and to bind non-canonical structures^16^. Finally, we also showed that *Tc*HMGB can act as an inflammatory mediator like other HMGB family members^17^. Then, we decided to perform functional studies for *Tc*HMGB in the parasite. The complete genome sequence of *T*. *cruzi* has been published in 2005 allowing genome-wide and *in silico* studies^18^. However, many biological aspects of this parasite remain unveiled due to its unusual characteristics and genome complexity and because the available tools for genetic manipulation of *T*. *cruzi* are relatively scarce, particularly compared to other members of the trypanosomatid family, such as *Trypanosoma brucei*^19,20^. The toolkit available to be used in *T*. *cruzi* research is limited to a low number or episomal and integrative constitutive expression vectors and the tetracycline (Tet)-inducible system based on plasmid p*Tc*INDEX^21^. The RNA interference system is not active in *T*. *cruzi* and gene knock out by homologous recombination is very inefficient. Recently, CRISPR/Cas9 nuclease system has been used to disrupt several genes in *T*. *cruzi*, but it cannot be used to obtain stable null mutants of essential genes^22–24^. In fact, obtaining stable knock-outs may be almost impossible even if the gene of interest is not completely essential but its deletion confers a selective disadvantage or impaired growth preventing knock out selection.

Therefore, in the present work we used the p*Tc*INDEX-GW inducible vector (a GATEWAY^®^ Cloning Technology-adapted p*Tc*INDEX version built in our laboratory) to continue studying *Tc*HMGB *in vivo*^25^. Data showed that *Tc*HMGB can alter the nuclear structure in *T*. *cruzi* epimastigotes and seems to be important for fundamental processes like replication, cell cycle progression, infection and metacyclogenesis. Overexpression of *Tc*HMGB caused a marked decrease in epimastigotes growth and an accumulation of parasites in G2/M phase, probably associated to a failure in cytokinesis. Also, we observed a decrease in the infection rate in Vero cells, amastigotes proliferation and the number of trypomastigotes released from infected cells *in vitro* in *Tc*HMGB-overexpressing parasites. These results suggest that, as well as other HMGB family members, the *T*. *cruzi* HMGB can be considered as a pleiotropic factor involved in key cellular processes that may play a role in Chagas disease pathogenesis.

## Results

### Nuclear ultrastructure and chromatin state are affected by *Tc*HMGB protein levels

To gain insight into the *Tc*HMGB protein function *in vivo*, we constructed parasite strains capable of overexpressing *Tc*HMGB. We used the inducible vector p*Tc*INDEX-GW^21,25^ to obtain transgenic parasites expressing the protein fused to a C-terminal double hemagglutinin tag (HA)_2_ under the control of a Tetracycline (Tet)-inducible promoter. Overexpression after Tet-induction was tested by Western blot and qRT-PCR, showing approximately 12-fold protein overexpression and ∼200 change fold in mRNA levels relative to non-induced control in epimastigotes (Fig. S1A,B). We also analyzed the plasmid-derived protein expression by western blot with antibodies directed to the HA-tag and confirmed that *Tc*HMGB protein is overexpressed in all life cycle stages and there is almost no leaky expression in the absence of tetracycline (Fig. S1C). To rule out possible side-effects of the overexpression induction, we constructed a control strain that, under the same conditions, overexpresses a mutant *Tc*HMGB that bears the two HMGB-box domains characteristic of the HMGB family but lacks the N-terminal domain necessary to direct the protein to the nucleus (Dm28c/p*Tc*INDEX-GW-ΔN-*Tc*HMGB(HA)_2_) (see Fig. S2).

**Figure 1:**
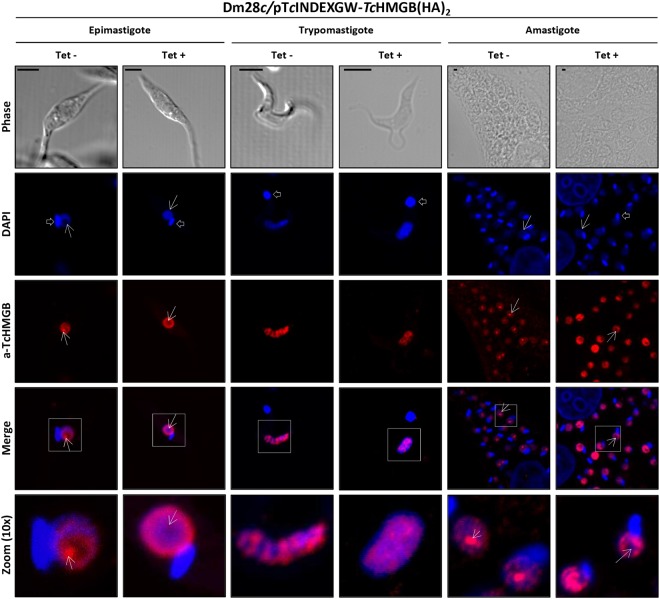
*Tc*HMGB overexpression affects nuclear structure and the protein localization. Confocal Immunofluorescence microscopy of non-induced (Tet−) and induced (Tet+) (0.5 μg/ml tetracycline, 12 h) *T*. *cruzi* Dm28c/p*Tc*INDEX-GW-*Tc*HMGB(HA)_2_ epimastigotes, trypomastigotes and amastigotes. The nucleus and kinetoplast were labeled with DAPI (blue); rabbit anti-*Tc*HMGB (a-*Tc*HMGB) was revealed with Cy3-conjugated anti-rabbit IgG antibodies (red). Arrows indicate the nucleolar region and open arrows the kinetoplast. Scale bar: 1 μm.

Like other HMGB proteins, included the endogenous *Tc*HMGB^16^, the plasmid-derived *Tc*HMGB(HA)_2_ was directed to the nucleus, giving a strong nuclear signal by immunofluorescence confocal microscopy both using specific antibodies and commercial anti-HA monoclonal antibodies in all the parasite life cycle stages (Fig. S1D). In contrast, the truncated protein localized in the cytoplasm as expected (Fig. S2D). Surprisingly, despite keeping the nuclear localization, *Tc*HMGB showed a different labeling pattern after Tet-induction, being particularly evident in epimastigote and amastigote forms (Fig. 1). Under normal (non-induced) conditions, *Tc*HMGB appears as a strong signal localized in the nucleolus accompanied by other spots of lower intensity distributed through the nucleoplasm in both replicative forms. In contrast, in Tet-induced *Tc*HMGB-overexpressing epimastigotes, the protein concentrates adjacent to the nuclear envelope and appears to be excluded from the nucleolus. Similarly, in overexpressing amastigotes, *Tc*HMGB label is seen as fluorescent spots of similar intensity regularly distributed along the whole nucleus except for the nucleolar region, from where the protein seems to be also excluded. In trypomastigotes, the non-replicative stage, there is no defined nucleolar structure and *Tc*HMGB is more regularly distributed through the nucleus. However, the protein localization also changed after Tet-induction: while in non-induced trypomastigotes *Tc*HMGB concentrates in discrete nuclear territories (regularly distributed in the nucleus), the fluorescent label appears more diffuse in overexpressing parasites.

It is worth noting the apparent relationship between *Tc*HMGB and the nucleolus, whose primary function is rRNA transcription and ribosomes assembly. The nucleolus can be easily identified in DAPI (4′,6-diamidino-2-phenylindole)-stained cells as the nuclear region without labeling, just because this region is essentially composed by ribonucleoproteins. Interestingly, this nuclear subdomain appears to be reduced in *Tc*HMGB-overexpressing epimastigotes (Fig. 1).

**Figure 2:**
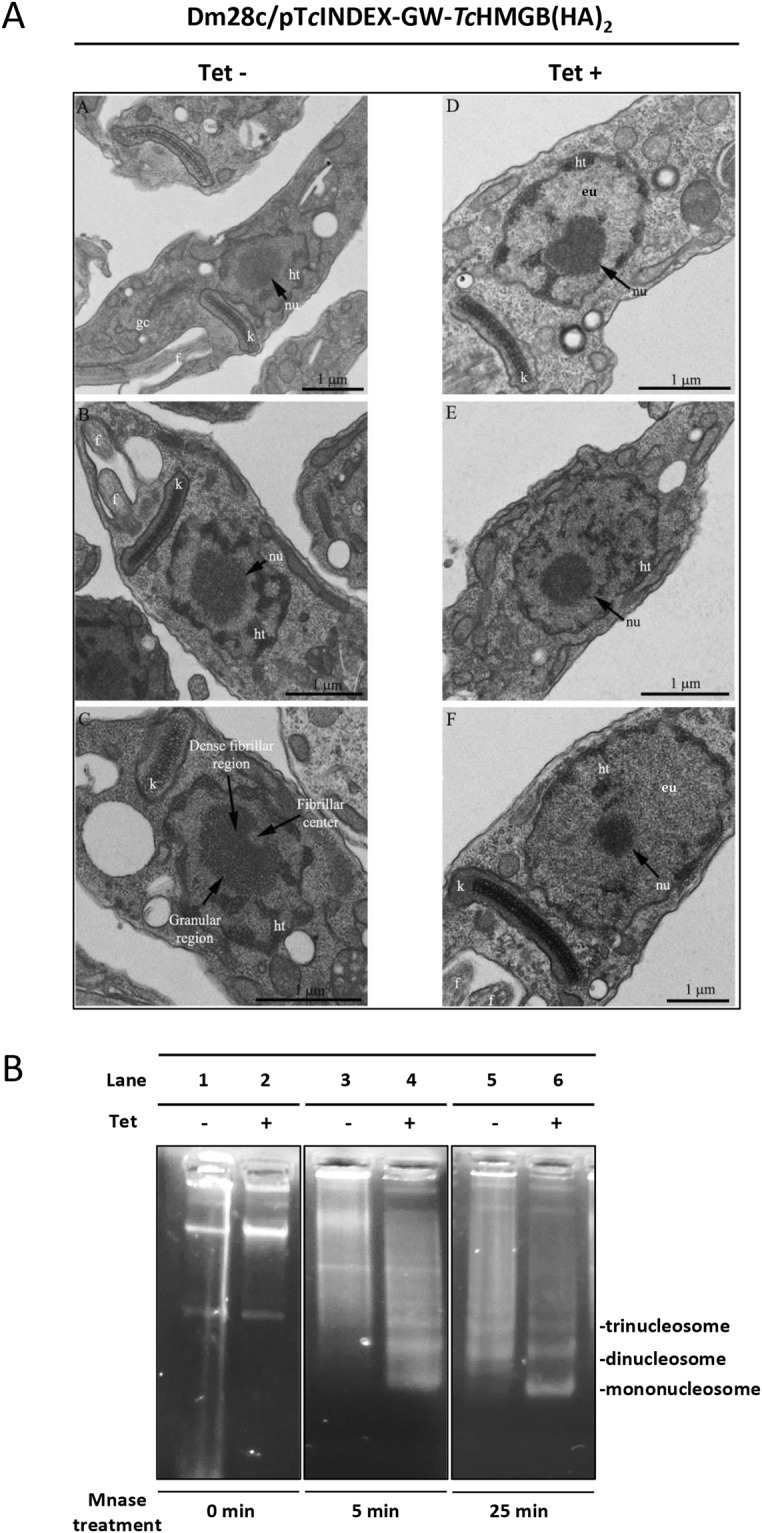
Nuclear ultrastructure and chromatin state are affected by *Tc*HMGB protein levels. (**A**) Transmission Electron Microscopy (TEM) analysis of the ultrastructure of the *T*. *cruzi* Dm28c/p*Tc*INDEX-GW-*Tc*HMGB(HA)_2_ epimastigotes in the absence (Tet−, Panels A–C) or presence (Tet+, Panels D–F) of tetracycline. Note the augmented space occupied by euchromatin (eu) and the reduction of the nucleolus in induced (Tet+) (**E,F**) in relation to non-induced parasites (Tet−) (**B**,**C**). Arrows indicate the nucleolus and in panel C its distinct domains. Kinetoplast (k), flagellum (f), Golgi complex (gc) and the different nucleolus regions are also indicated. (**B**) Analysis of chromatin isolated from *T*. *cruzi* Dm28c/p*Tc*INDEX-GW-*Tc*HMGB(HA)_2_ epimastigotes non-induced (−, lanes 1, 3, 5) or induced (+, lanes 2, 4, 6) with tetracycline (Tet). Chromatin from an equal number of cells was isolated and digested with 1 unit of micrococcal nuclease (MNase) for 0 (lanes 1, 2), 5 (lanes 3, 4) and 25 minutes (lanes 5, 6). Equal amounts of DNA were loaded on an ethidium bromide-stained 1% agarose gel. Note that in Tet−induced parasites, the characteristic ladder pattern is observed at shorter times compared to non-induced (Tet−). A representative experiment is shown, digestion products corresponding to DNA that has been bound to mono- di- and tri-nucleosomes are indicated. The corresponding full-length gel is presented in Supplementary Fig. S4.

Transmission electron microscopy (TEM) images of Tet-induced parasites (Fig. 2A panels D to F), also showed a reduction of the nucleolus (nu) associated to *Tc*HMGB overexpression, when compared to non-induced parasites (Fig. 2A panels A to C). This nucleolar reduction was confirmed by area measurements performed on TEM images (Table 1). In relation to non-induced (Tet−) parasites, the overexpressing epimastigotes (Tet+) presented a 28% increase in the total nuclear area. Considering nuclear domains, the euchromatin (eu) area augmented in 109%, whereas the nucleolus was reduced in a 55%. The heterochromatin (ht) area remained the same in both cell types, suggesting that the increase value obtained for euchromatin is not due to DNA unpacking, but results from nucleolar disorganization and the increase in the total nuclear area.

**Table 1:** Measurements of the total nucleus area and nucleolar domains in µm^2^.

Measured area (μm^2^)	Nucleus	Nucleolus	Heterochromatin	Euchromatin
Tet−	1.753 ± 0.112	0.475 ± 0.299	0.590 ± 0.037	0.687 ± 0.102
Tet+	2.245 ± 0.109	0.215 ± 0.073	0.590 ± 0.086	1.440 ± 0.182
Percentage change	+28%	−55%	—	+109%
Statistically different?	Yes	Yes	No	Yes

One hundred cells were measured in each one of three independent experiments. Statistics were calculated using the unpaired t test with Welch’s correction in GraphPad Prism 6 software. P values less than 0.05 were considered statistically significant.

Afterwards, we investigated the effect of *Tc*HMGB overexpression on the chromatin structure using micrococcal nuclease enzyme (MNase). MNase preferentially cleaves the double stranded naked DNA located between nucleosomes, thus giving a typical ladder pattern corresponding to mono-, di-, tri- and so on -nucleosomal species in DNA gel electrophoresis (Fig. 2B). A more relaxed or open chromatin structure is more accessible to the enzyme and consequently more prone to be digested than highly condensed chromatin. Accordingly, we verified that overexpression of *Tc*HMGB after Tet-induction results in a chromatin more sensitive to MNase treatment evidenced by the typical ladder pattern obtained at shorter times compared to non-induced control. Indeed, after a 5 minute-treatment, the smallest band corresponding to one nucleosome-DNA is already visible in the induced sample (Fig. 2B, lane 4) while, in the non-induced control, longer times are required to observe a similar pattern (Fig. 2B, lane 5). The enrichment in smaller fragments and disappearance of larger DNA molecules after 25 min treatment in the induced sample (Fig. 2B, lane 6) means that the MNase was able to reach more spacer DNA between nucleosomes. This data is in accordance with the increased area of euchromatin observed by TEM in *Tc*HMGB overexpressing cells.

### *Tc*HMGB overexpression decreases cell proliferation and affects cell division and morphology of epimastigotes

**Figure 3:**
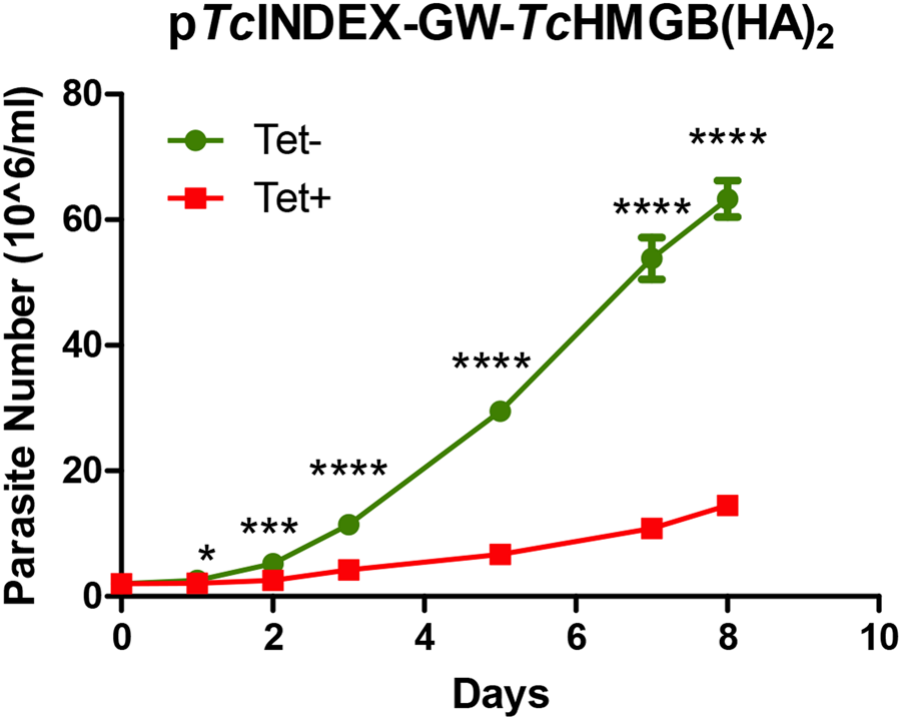
*Tc*HMGB overexpression decreases cell proliferation. Growth curve of epimastigotes transfected with p*Tc*INDEX-GW-*Tc*HMGB(HA)_2_ in the absence (green circles, Tet−) or presence (red squares, Tet+) of 0.5 µg/ml tetracycline, counted daily for 8 days. Values represent mean ± SD of 3 independent replicates, *p < 0.05, ***p < 0.0005, ****p < 0.00005 (Student t test).

The *Tc*HMGB overexpression clearly affected epimastigote growth, as can be seen in Fig. 3. Indeed, as soon as 24 hs post-induction, the parasite number appears reduced in induced cultures compared to the non-induced ones. Even though the difference seems minimal at this time point (2.12 × 10^6^ vs 2.58 × 10^6^ parasites/ml, that is only 0, 46 parasites/ml difference), it is already statistically significant. *Tc*HMGB-overexpressing epimastigotes proliferation was slower in relation to control (non-induced) cells during the whole growth curve, increasing the difference in the parasite number with time. At day 2 post-induction (pi), the average difference in the number of parasites was 2.7 × 10^6^ parasites/ml, reaching 43 × 10^6^ parasites/ml at day 7 pi (comparing the mean values in each time point). This growing difference was not seen after Tet-treatment of wild type parasites or those transfected with p*Tc*INDEX-GW-GFP (Fig. S3) or other constructions in the same p*Tc*INDEX vector^25–28^. Regarding the overexpression of the mutant *Tc*HMGB, although we observed a slight delay in the growth curve, the duplication time of the induced vs. non-induced parasites, doesn’t show a significant difference until the fifth day post-induction and the difference in parasite number in each time point is low during the whole period (0.24 × 10^6^ parasites/ml at day 2 pi to 5 × 10^6^ parasites/ml at day 7 pi) (Fig. S2). This data suggests that the higher levels of *Tc*HMGB inside the nucleus and the associated changes in the nuclear and chromatin structure are responsible for the growing defect observed for *Tc*HMGB-overexpressing epimastigotes. In contrast, the observed slight reduction in the ΔN-*Tc*HMGB-overexpressing epimastigotes growth could be associated to non-nuclear functions of *Tc*HMGB, similar to those described for other HMGB family proteins orthologs, not yet studied in *T*. *cruzi*^29,30^.

**Figure 4:**
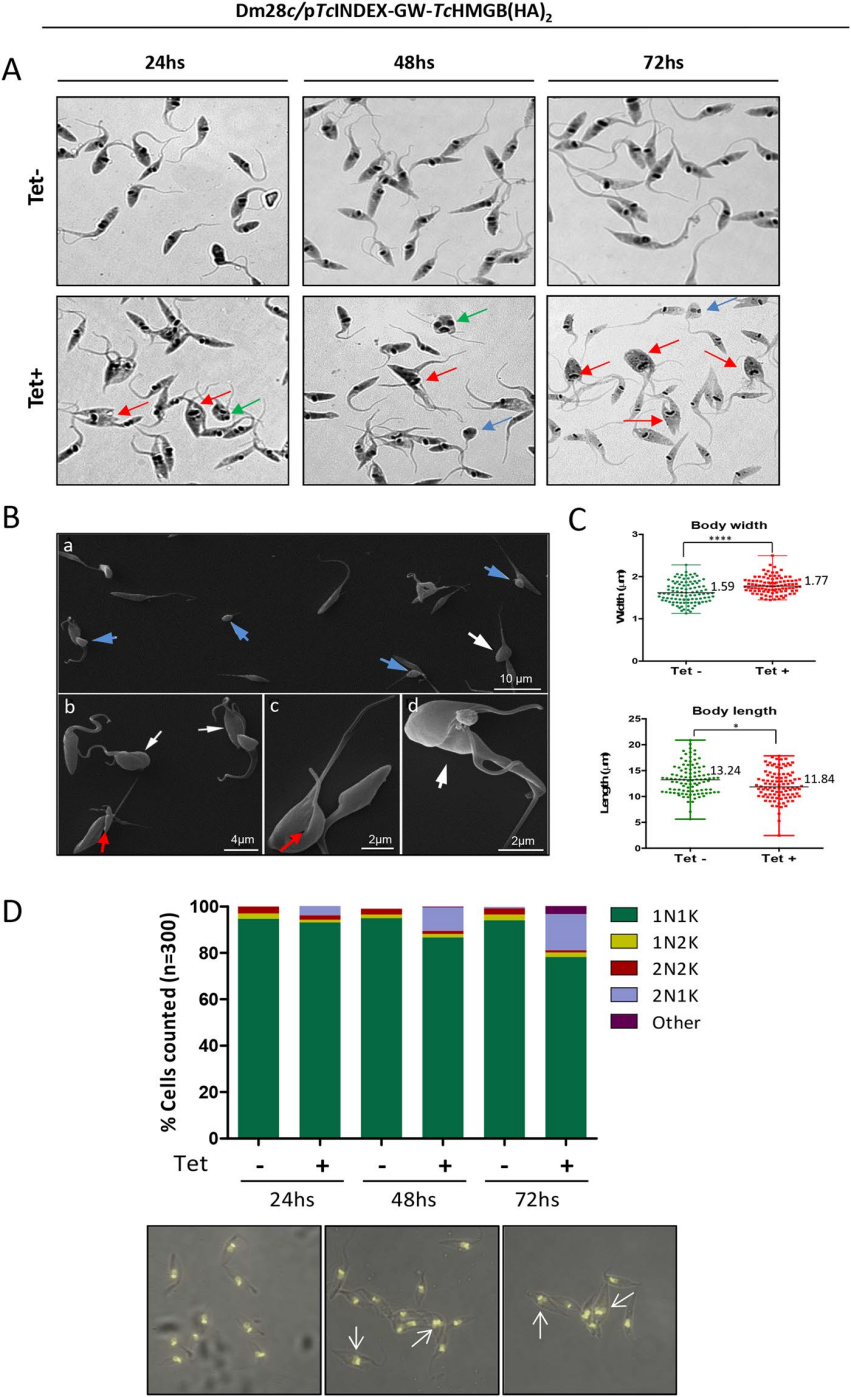
*Tc*HMGB overexpression affects cell morphology and division. (**A**) May Grünwald Giemsa-stained *T*. *cruzi* Dm28c/p*Tc*INDEX-GW-*Tc*HMGB(HA)_2_ epimastigotes in the absence (−) or presence (+) of Tet at 24, 48 and 72 h post-induction. Red arrows indicate parasites with apparent impaired cytokinesis. Some of them present altered cell morphology for example, two nuclei and one kinetoplast (green arrows) and reduced sizes (blue arrows). (**B**) Images obtained by Scanning Electron Microscopy (SEM) showed that the induction of *Tc*HMGB overexpression promoted the appearance of cells with reduced sizes (panel a, blue arrows) and a high number of parasites in cytokinesis, presenting an enlargement or widening of the cell body (white arrows). Some cells presented an asymmetric division (panels b and c, red arrows), which can generate parasites with reduced sizes (panel a, blue arrows). (**C**) Analysis of the parasite body dimensions. The body widths and lengths of control (Tet−) and induced (Tet+) epimastigotes were measured from the SEM images. Wilcoxon-Mann-Whitney Test confirmed that the values for width and length between the (Tet−) and (Tet+) groups are significantly different (p < 0.05), N = 101, Dot plot graphs represent individual values, median and range are indicated; *p < 0.05, ****p < 0.0001. (**D**) Nucleus/Kinetoplast content (N/K) of Dm28c/p*Tc*INDEX-GW-*Tc*HMGB(HA)_2_ epimastigote cultures in the absence (Tet−) or presence (Tet+) of tetracycline at different time points (24, 48 and 72 hours). Data from two independent experiments were considered in the analysis (n = 300 cells for each column). Below, representative pictures of the microscopy images analyzed to perform the graph. White arrows show examples of epimastigotes with 2 segregated nuclei and 1 kinetoplast (2N1K).

Interestingly, Tet-induced *Tc*HMGB overexpressing parasites also showed morphological differences when compared to non-induced epimastigotes according to data obtained by optical and scanning electron microscopy (SEM). For example, we observed an increasing number of parasites where the kDNA has been duplicated, but the kinetoplast has not been segregated, thus impairing the cytokinesis (Fig. 4A, red arrows). Parasites with aberrant morphologies or reduced dimensions were also observed (Fig. 4A,B, blue arrows), as well as a population of epimastigotes with two nuclei and only one kinetoplast (Fig. 4A, green arrows; Fig. 4D, white arrows). By SEM, it was also possible to identify *Tc*HMGB overexpressing cells in cytokinesis (Fig. 4B, panels b and d, white arrows) or undergoing asymmetric divisions (Fig. 4B, red arrows on panels b and c). Finally, a quantitative analysis of the parasite measures from SEM images showed that an increased proportion of *Tc*HMGB overexpressing cells presented a slight decrease in length and a small increase in the width of the cell body, which may be related to division impairment (Fig. 4C).

The ordered progression of the cell cycle, in which kinetoplast segregation precedes nuclear division, allows the identification of three normal states regarding nuclear/kinetoplast (N/K) content: 1N1K, 1N2K and 2N2K^31^. Under normal conditions, most epimastigotes in a non-synchronous exponentially growing culture contain one nucleus and one kinetoplast (1N1K, usually ∼80–95%), corresponding to parasites in G1 or S phase of the cell cycle^32^. A smaller proportion exhibits two kinetoplasts and one nucleus (1N2K ∼2.3%), these correspond to parasites in G2 phase or the beginning of mitosis. Finally, cells presenting two kinetoplasts and two nuclei (2N2K ∼3%) are those that have finished mitosis and are undergoing cytokinesis or ready to do so. Thus, the appearance of cells with abnormal N/K content (for example, 0N1K, 1N0K or 2N1K) is indicative of a possible cell cycle deffect^31^. In fact, after analyzing the N/K content of the *Tc*HMGB-overexpressing epimastigotes, we found an increase of 5% of cells presenting the phenotype 2N1K, 24 hs post-induction. This percentage augmented with time, reaching approximately 18% of the total population after 72 hs of tetracycline induction. These data are in accordance to cytokinesis impairment and suggests a failure in the cell cycle progression in *Tc*HMGB overexpressing parasites (Fig. 4D).

### *Tc*HMGB overexpression alters cell cycle progression

**Figure 5:**
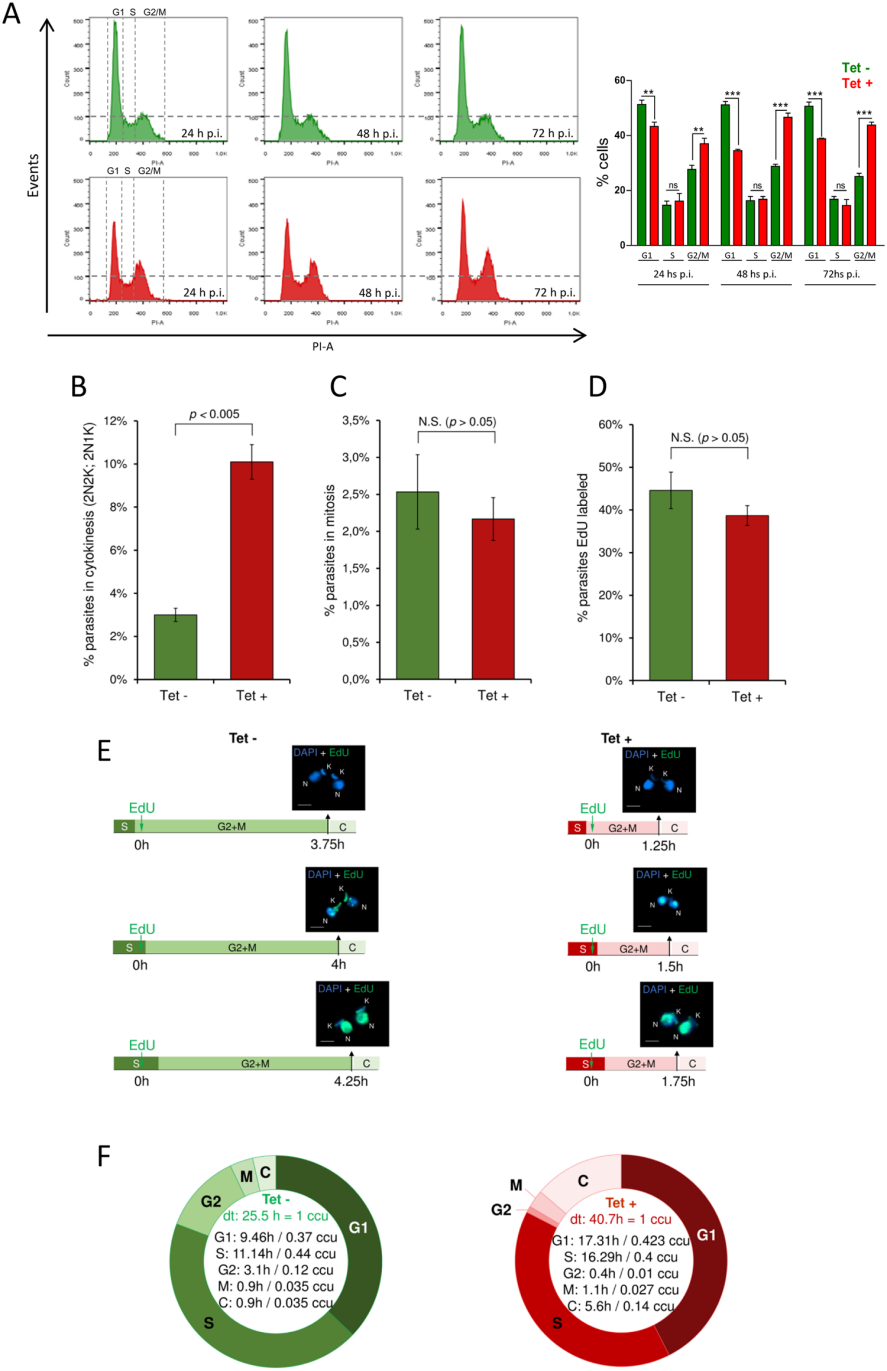
*Tc*HMGB overexpression alters cell cycle progression. (**A**) Flow Cytometry analysis of the cell cycle progression. On the left, Flow Cytometry analysis of cultured *T*. *cruzi* Dm28c/p*Tc*INDEX-GW-*Tc*HMGB(HA)_2_ epimastigotes at different times post-tetracycline induction (p.i.). Histograms are plotted as number of events vs. propidium iodide absorbance (PI-A). On the right, bar graph with the percentages of cells in the different phases of the cell cycle. **p < 0.005, ***p < 0.0001 (Student t test). (**B–F**) Estimation of the duration of each cell cycle phase. *T*. *cruzi* Dm28*c*/p*Tc*INDEX-GW-*Tc*HMGB(HA)_2_ epimastigotes in exponential phase of culture induced (Tet+) or not (Tet−) with tetracycline, were used in these analysis. (**B**) DAPI-labeled parasites (2N2K and 2N1K) were used to measure the percentage of parasites in cytokinesis, which was estimated to be 3.0% ± 0.3 for Tet− group (green) and 10.1% ± 0.8 for Tet+ group (red). Error bars represent SD. The values shown represent the average of three independent assays. These values were used in Williams (1971) equation to estimate the duration of cytokinesis phase. (**C**) Parasites with nuclei in division but not yet segregated were used to estimate the proportion of parasites performing mitosis [2.5% ± 0.5 for Tet− (green), and 2.2% ± 0.2 for Tet + (red)]. Error bars represent SD. The values shown represent the average of three independent assays. These values were used in Williams (1971) equation to estimate the duration of mitosis phase. (**D**) Parasites EdU-labeled after 1 h of EdU pulse were used to estimate the percentage of parasites replicating DNA [44.6% ± 4.3 for Tet– (green), and 38.7% ± 2.3 for Tet+ (red)]. Error bars represent SD. The values shown represent the average of three independent assays. These values were used in Stanners and Till (1960) equation to estimate the duration of S phase. (**E**) To estimate the duration of G2 + M phases, the thymidine analog EdU was added to the culture and parasites were collected every 15 min until parasites containing two EdU-labeled nuclei were observed (2N2K or 2N1K). In Tet– (green), this pattern was observed after 4 h, and in Tet + (red) after 1.5 h. This assay was carried out in triplicate and in all of them, we found a parasite containing two EdU-labeled nuclei at the same time. The scale bar on the fluorescence images corresponds to 2 µm. (**F**) Schematic representation showing the duration of each cell cycle phase established using EdU. Of note, ccu means cell cycle unit, where one unit corresponds to the specific doubling time (dt) for each strain. The statistical analysis for Fig. 5B-D was made with Student t test using GraphPad Prism 6. As expected, the significant statistical difference (comparing Tet− and Tet + ) relative to parasites performing cytokinesis (Fig. 5B) is reflected in the cytokinesis phase lengths estimated for both Tet− and Tet + (Fig. 5F). Of note, there was no significant statistical difference between parasites EdU-labeled or parasites performing mitosis (p > 0.05).

The cell cycle progression of *Tc*HMGB overexpressing epimastigotes was analyzed by flow cytometry with Propidium iodide (PI) staining for 72 h post-induction and compared with non-induced control parasites (Fig. 5A). As expected, in the absence of Tet we did not observe major changes with time in the counts for each population. The main peak corresponds to the parasites on G1 phase of the cell cycle (∼50% of the total), that is, parasites with the DNA content corresponding to one nucleus (2n). A second minor peak represents the parasites in G2/M phase (∼25–28%), which corresponds to epimastigotes with the double of DNA content (4n), including those on cytokinesis. In the valley between the two peaks are the cells on S phase (∼15%). As can be seen in the lower (red) panel of Fig. 5A, the peak of cells in G2/M increased after Tet-induction ranging from 38% at 24 post-induction to 48% at 48–72 h (Fig. 5A), suggesting that the *Tc*HMGB overexpression results in an arrest of cells containing duplicated DNA (4n). Our hypothesis, in accordance with the previous microscopy observations, is that cytokinesis is impaired in overexpressing parasites and causes the accumulation of parasites in the G2/M peak, which in fact includes parasites that have not finished the cell division.

Afterwards, we made use of DAPI and the thymidine analog EdU (5-Ethynyl-2´-deoxyuridine) to follow the cell cycle progression at the microscope and estimate both the duration and the percentage of parasites in each cell cycle phase^33^. In accordance to our previous results, we confirmed using DAPI staining the presence of an increased percentage of parasites in cytokinesis presenting typical (2N2K) or atypical (2N1K) cellular patterns (Fig. 5B). Parasites with nuclei in division but not yet segregated were used to estimate the proportion of parasites performing mitosis, which did not show major differences compared to non-induced parasites (Fig. 5C). Finally, the thymidine analog EdU was added to the culture and parasites labeled after 1 h were used to estimate the percentage of cells replicating DNA (Fig. 5D). The duration of cytokinesis and mitosis phases were estimated through Williams equation^34^, using the percentage of parasites in each respective phase. To estimate the duration of G2/M phases, we made an EdU 1h-pulse and then collected parasites every 15 min in order to observe parasites containing two EdU-labeled nuclei (2N2K or 2N1K) (Fig. 5E). In non-induced (Tet−) cultures, this N/K pattern was observed after 4 h, while in Tet-induced (Tet+) after 1.5 h. The difference between this value and the duration of mitosis previously calculated by Williams equation^34^ corresponds to G2 phase duration. The duration of S phase was estimated according to the Stanners and Till equation^35^. Figure 5F shows a schematic representation of the duration of each cell cycle phase. The whole cycle lasts 40.7 h in *Tc*HMGB overexpressing parasites culture versus 25.5 h for the non-induced control culture. This difference in duplication times for the induced vs non-induced parasites is consistent with the delay in the growth curve described previously (Fig. 3). Also, as expected, the cytokinesis lasts longer for the Tet−induced culture, contributing to the whole cell cycle duration, together with the G1 phase, which is actually estimated by the difference between the duplication time and the estimated times for the other phases. The S phase lasts 16.29 h for Tet+ vs. 11.14 h for Tet−. The M phase showed no great differences, but it is noteworthy the shortening in G2 for Tet+. We must emphasize that the DNA content of cells in G2, M, or cytokinesis is exactly the same (4n). Thus, G2 + M + C takes 2.2 h more hours for the *Tc*HMGB overexpressing parasites, supporting the accumulation of 4n-containing cells observed by flow cytometry in the *Tc*HMGB overexpressing parasites. Together, our data support the hypothesis of an impaired or delayed cytokinesis caused by *Tc*HMGB overexpression.

### *Tc*HMGB levels can affect cell infection, amastigote replication and metacyclogenesis

To investigate if *Tc*HMGB can be important for Chagas disease pathogenesis, we needed to focus on the parasite life cycle stages present in the mammalian host. As a first approach, we analyzed *in vitro* the performance of transgenic parasites overexpressing *Tc*HMGB to invade and replicate inside the host cells. We used different Tet-induction schemes to analyze the *Tc*HMGB-overexpression effect over the different time points during the whole *in vitro* infection process (see Methods section).

**Figure 6:**
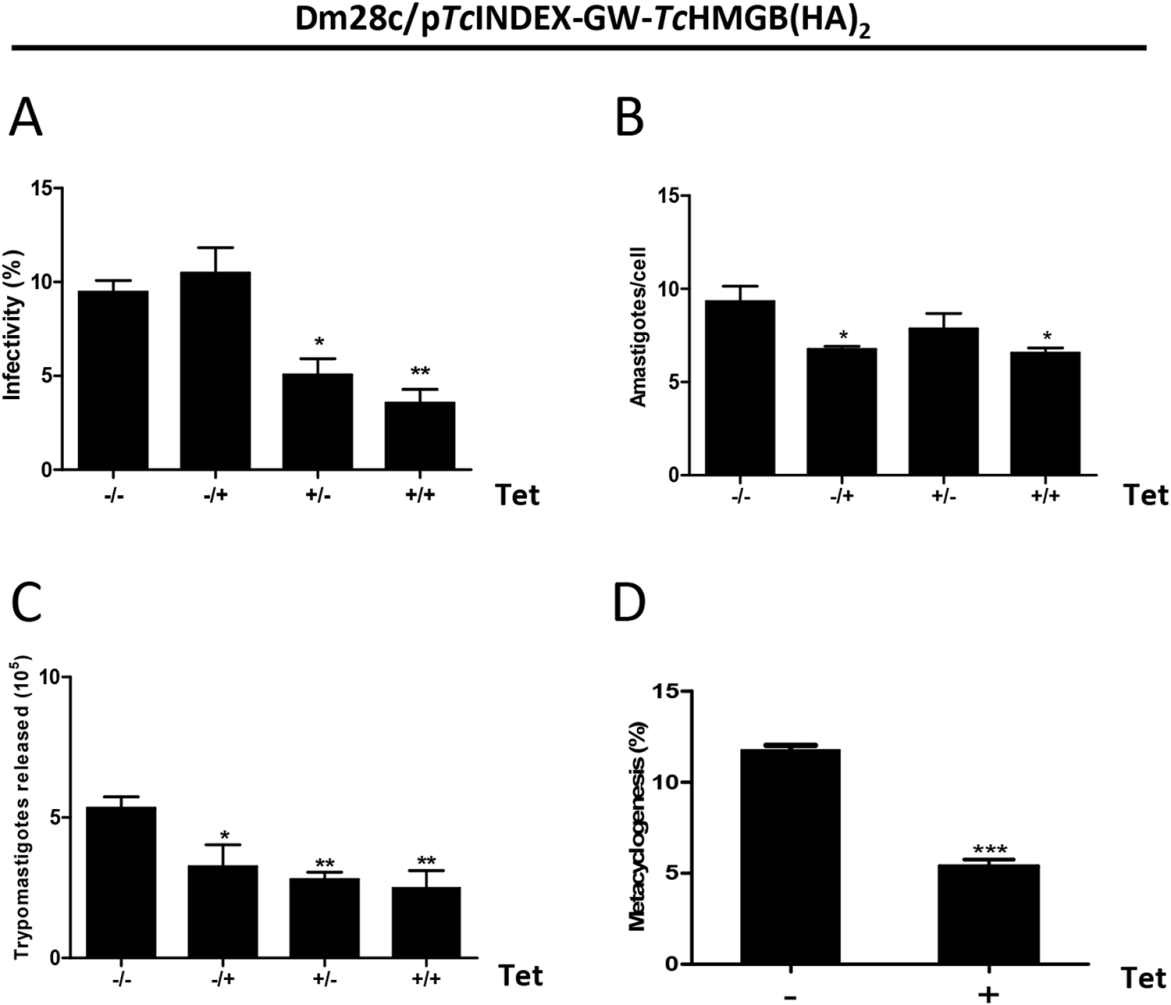
*Tc*HMGB levels affect cell infection, amastigote replication and metacyclogenesis. The infection performance of *T*. *cruzi* Dm28c/p*Tc*INDEX-GW-*Tc*HMGB(HA)_2_ was analyzed in the absence (Tet−) or presence (Tet+) of 0.5 μg/ml tetracycline. To test the effect over the different phases of infection, we designed different induction protocols as follows: (−/−), Tet was never added to the medium, non-induced control; (+/−), trypomastigotes were pre-treated with Tet for 2 hours prior to infection, and during the invasion incubation period but not after; (−/+), trypomastigotes were not induced, Tet was only added after 48 hours post-infection to see the effect on the amastigote stage; (+/+), trypomastigotes were pre-treated and Tet was present during the whole infection assay, to see the overexpression effect both in trypomastigotes and amastigotes. The percentage of infected cells (**A**), the number of amastigotes per cell (**B**) and the number of trypomastigotes released 6 days post-infection (**C**) were determined by counting Giemsa-stained slides using an optical microscope. Results are expressed as mean ± SD of triplicates. Statistical analysis of the data was carried out using one-way ANOVA, *p < 0.05, **p < 0.001 and ***p < 0.0005. (**D**) *In vitro* metacyclogenesis using TAU medium of the p*Tc*INDEX-GW-*Tc*HMGB(HA)_2_ parasites non-induced (−) or induced (+) with 0.5 µg/ml Tet for 96 h. The bar graph represents the mean ± SD from three independent experiments; ***p < 0.0005 (Student t test).

To study if trypomastigote ability to invade and infect cells on a monolayer was affected by *Tc*HMGB overexpression, trypomastigotes were pre-incubated with Tet (Fig. 6, +/− and +/+ condition) or not (−/+ and −/− condition) and then allowed to infect a monolayer of Vero cells. After 6 hs infection, free trypomastigotes were washed out and fresh medium with (+/+ and −/+ condition) or without Tet (+/− and −/− condition) was added to the cells and incubated for 3 days. The −/− condition represents the non-induced control where Tet was never added and the +/+ condition shows the effect of protein overexpression during the whole infection assay (from prior to invasion “+/” and through the 3 days left for amastigote replication inside the host cell “/+”). The trypomastigotes infection performance was analyzed by the proportion of infected Vero cells (Fig. 6A) and intracellular amastigotes replication ability was measured as the average number of intracellular amastigotes per infected cell at 72 hs post-infection (Fig. 6B).

Overexpression of *Tc*HMGB on trypomastigotes by pre-treatment with Tet before infection caused a dramatic decrease (∼52%) in the infection rate (Fig. 6A, compare +/− and +/+ conditions with −/−). As expected, the addition of Tet after the incubation period to allow trypomastigotes invasion of Vero cells (−/+), showed no significant difference in the proportion of infected cells compared to the negative control (−/−). In contrast, amastigote duplication was affected in this condition (−/+). As can be seen in Fig. 6B, the addition of Tet once the trypomastigotes have entered the host cells (−/+) or when Tet was always present (+/+), caused a reduction in the average number of intracellular amastigotes per cell (compared to −/−) showing an effect on this parasite stage too. Furthermore, the number of trypomastigotes released to the supernatant at day 5 post-infection due to the lysis of infected cells (Fig. 6C), was also lowered in *Tc*HMGB-overexpression conditions, either when Tet was added only to pre-incubate trypomastigotes (+/−), only to intracellular amastigotes (−/+) or when it was always present (+/+). The number of released trypomastigotes is influenced by the infectivity of trypomastigotes and by the amastigotes replication rate, but also the efficiency in the transformation between the different life cycle forms (from trypomastigote to amastigote and back to the trypomastigote form finally released) may contribute to the final number of released parasites. It is difficult to assess how much each of these processes affects the final number of released trypomastigotes, in particular it is not easy to measure the transformation between the parasite life cycle stages contribution. However, we can easily evaluate *in vitro* the epimastigote to metacyclic trypomastigote transformation process to see if it is affected by *Tc*HMGB overexpression. Indeed, *in vitro* metacyclogenesis was performed in the absence or presence of Tet, and *Tc*HMGB overexpression resulted in a reduced number of metacyclic trypomastigotes (Fig. 6D).

## Discussion

As in other eukaryotic cells, nuclear architecture is dynamic in trypanosomes. Chromatin shows differences through the parasites life cycles and also through cell cycle in proliferating stages regarding its position inside the nucleus, condensation state and protein composition^3,36,37^. These changes thus modify DNA accessibility to protein complexes involved in nuclear functions like transcription and replication. The histones suffer different post-translational modifications through the parasite life cycle, such as methylation or acetylation, that contribute to chromatin remodeling and epigenetic control of gene expression^38^. Several epigenetic control mechanisms have been already described in trypanosomatids^39–44^. Interestingly, it has recently been reported that chaetocin, a histone methyltransferases inhibitor, promoted irreversible inhibition of protozoa growth presumably as a consequence of the unpacking of nuclear heterochromatin and intense nucleolus fragmentation, which is associated with the parasite cell cycle arrest and RNA transcription blockage^45^. It is clear that chromatin structure must be well regulated for an accurate performance of the parasite functions, however, there are still many unsolved issues regarding how these parasites control their gene expression and how chromatin and nuclear structure changes are achieved during the parasite differentiation.

High mobility group B proteins are important players in chromatin structure. These proteins have shown to facilitate chromatin remodeling thus increasing accessibility of the nucleosomal DNA to transcription factors and protein complexes involved in transcription, replication, recombination, DNA repair and genomic stability^9^. TDP1, the ortholog HMGB from *T*. *brucei*, showed to be directly involved in epigenetic control of RNA Polymerase I (RNAP I)-transcription of rDNA and VSG genes^43,46^. In our previous study, we demonstrated that *Tc*HMGB is an architectural protein capable of modifying DNA structure *in vitro*^16^. Here, we attempted to study the putative roles of the protein in the parasite. Protein overexpression is a valuable approach^25,28,47^, considering that silencing by RNAi is not possible in *T*. *cruzi*, and our failure to obtain knockout parasites, thus suggesting that the gene may be essential or, at least, that its deletion results in a growth disadvantage. Using a p*Tc*INDEX-GW vector-derived construction, we were able to overexpress *Tc*HMGB in *T*. *cruzi*. Tet−induced parasites presented modifications not only in the cell morphology but also in its nuclear structure, diminishing its replication rate and affecting several functions related to the parasite biology, as infectivity and differentiation. It is worth mentioning that the observed behavior of *Tc*HMGB overexpressing parasites is due to the increased *Tc*HMGB protein in the nucleus where it can interact with the DNA since none of these phenomena were observed after overexpression of a truncated version of the protein, which has the two functional HMG-Box domains but localizes in the cytoplasm of the parasites (Fig. S2).

Taking into account the architectural properties of HMGB protein family and our previous *in vitro* evidence, it was expected that *Tc*HMGB interaction with genomic DNA would also occur *in vivo*^16^. Thus, an altered chromatin structure would be expected in parasites with an increased content of this protein in their nuclei. Not surprisingly, the chromatin in these overexpressing parasites showed to be in a more relaxed or open state, as determined by its MNase sensitivity, suggesting that *Tc*HMGB can contribute to chromatin remodeling in the parasite. Accordingly, TEM analysis showed that higher *Tc*HMGB content results in a reduction of the nucleolus and an increase of the euchromatin region considering the total nuclear area. The nucleolus is the most evident nuclear domain and can be easily distinguished by microscopy techniques in eukaryotic cells. It is characterized by its resident proteins that play essential roles, as ribosomal RNAs transcription, processing and assemblage into ribonucleoprotein (RNP) that results in ribosome biogenesis. More recently, additional functions have been proposed for the nucleolus such us regulation of mitosis, cell cycle progression and stress response^48^.

Changes in nucleolar shape and size have been described in *T*. *cruzi* under stress conditions, like the induction of the stationary phase in cultured epimastigotes^49^ or when replicative forms transform into the non-proliferative trypomastigotes^3^. It is worth mentioning that these changes in the nucleus correlate with the parasite replication and transcription rates^50^. In transcriptionally active epimastigotes and dividing amastigotes the rounded nucleus contains the heterochromatin organized around the central nucleolus and in the nuclear periphery, while in trypomastigotes the nucleus is elongated, lacks an evident nucleolus and presents a disperse heterochromatin^3,4^. In our previous report, we showed that *Tc*HMGB is a nuclear protein expressed in all *T*. *cruzi* life cycle stages, although the protein content is higher in epimastigote and amastigote forms in comparison to the non-replicative stage^16^. The reduced *Tc*HMGB content in trypomastigotes correlates with increased amounts of heterochromatin and may be associated to their lower transcriptional activity. In accordance to this idea, the overexpression *Tc*HMGB would contribute to relaxation of chromatin, increasing the euchromatin in the nucleus and thus affecting different nuclear functions.

Regarding the observed reduction of the nucleolus, it is interesting to note the *Tc*HMGB immunofluorescence microscopy labeling pattern changes in overexpressing parasites. In wild type and non-induced parasites, *Tc*HMGB signal appears localized in the whole nucleus as several spots quite regularly distributed, except for a strong signal located in the nucleolus. Similarly, *T*. *brucei* TDP1, showed to be distributed throughout the nucleus in both bloodstream and procyclic forms, but enriched in either one or two discrete spots corresponding to the nucleolus and to specific expression site bodies (ESB) in bloodstream forms^46^. There, TDP1 presumably facilitates RNAP I dependent transcription of rRNA and VSG genes respectively^46^. Analogously, the strong signal of *Tc*HMGB in control cells may correspond to the rRNA transcription site in the *T*. *cruzi* nucleolus, whereas in overexpressing cells the redistribution of this protein could result from nucleolar disassembly or disorganization, as a consequence of higher production of rRNA transcripts. It is also worth noting that histone H1 showed to be depleted from the nucleolus in *T*. *brucei* presumably as a consequence of the extremely high rates of transcription of the ribosomal DNA (rDNA)^51^. In other organisms, HMGB proteins have shown to facilitate nucleosome remodeling and accessibility of the nucleosomal DNA through the displacement of histone H1. Thus, taking into account our observations and the evidence from homologous proteins, it seems likely that *Tc*HMGB can contribute to RNAP I-transcription of rDNA genes in *T*. *cruzi*. Finally, it cannot be ruled out that *Tc*HMGB may also contribute to the RNAPII-dependent transcription. In fact, RNAPII was found to concentrate in a domain close to the parasite nucleolus containing the spliced leader genes and the remaining protein was diffusely distributed in the nucleoplasm^52^, showing an immunofluorescence pattern similar to the one observed for *Tc*HMGB in wild type (or non-induced) parasites^16^.

When overexpression of *Tc*HMGB is induced by Tet, the protein appears more regularly distributed showing a strong immunofluorescence signal along the whole nucleus, but reduced or even excluded from the nucleolar region. This relocalization of *Tc*HMGB can be consequence of an ectopic aggregation of the protein as a consequence of the higher expression levels. This anomalous distribution of *Tc*HMGB may be responsible for the reduction or disassembly of the nucleolus observed by TEM and DAPI staining in overexpressing parasites. Indeed, the nucleolus is a dynamic structure and it is intimately linked to the processes that take place there and to their associated proteins. It has been already described that nucleolar markers are seen dispersed in the nucleoplasm when the nucleolus is reorganized during the life cycle or when dividing epimastigotes reach the stationary phase^49,53^. However, it is not clear which process is cause and which is consequence, that is, does nucleolar proteins redistribution cause the nucleolus to disassemble or *vice versa*? In other organisms, it has been shown that introduction of rDNA genes on non-integrating plasmids led to the formation of mini-nucleoli and inhibition of the RNAPI results in disassembly of nucleoli^54^. Thus, nucleolar proteins association and functions are linked to nucleolar structure formation.

If we assume that *Tc*HMGB concentrates in the nucleolus in replicating epimastigotes and amastigotes and facilitate rRNA transcription by maintaining an open chromatin state, it is possible that ectopically localized aggregates of the overexpressed protein results in the destabilization of nucleolar structure. Moreover, the overexpression of *Tc*HMGB and its anomalous localization in *T*. *cruzi* may be also interfering with the normal RNAP II transcriptional activity. Thus, we cannot rule out that a failure in the control of specific genes transcription may influence the observed phenotypes, since indeed all HMGB family members have pleiotropic functions. Additional work and a detailed study of both global and individual genes transcription in overexpressing parasites would be necessary to better understand the exact role of *Tc*HMGB in gene expression control. The overexpression of *Tc*HMGB can also interfere with proper DNA replication, thus affecting the normal development of the parasite functions. In fact, the replication origins have been localized at the boundaries of polycistronic transcription units suggesting a functional interaction between DNA replication and transcription initiation in *T*. *brucei* genome^55^, thus it is likely that an overexpression of *Tc*HMGB in the trypanosome nucleus causes an imbalance of both processes. This would explain the reduction in proliferation, the appearance of atypical phenotypes and cell cycle arrest in parasites where the *Tc*HMGB is displaced.

In *Tc*HMGB overexpressing cells, the destabilization of chromatin DNA structure might favor nuclear DNA replication, but this process would proceed uncoupled from kinetoplast DNA replication and cytokinesis. In accordance to this idea is the higher proportion of Tet-induced parasites presenting 2N1K and the delayed cytokinesis phenotype, which supports the hypothesis of a failure in DNA replication control. Somehow, this uncontrolled DNA replication may impair the proper progression of the cell cycle since when we analyzed the parasites by flow cytometry, we observed that *Tc*HMGB overexpression results in an accumulation of cells in G2/M phase of the cell cycle, which also includes parasites in cytokinesis. A higher number of cells in cytokinesis were observed by optical microscopy and SEM, corroborating the idea that this process was impaired in Tet-induced parasites. Furthermore, a detailed analysis of the cell cycle revealed that epimastigotes overexpressing *Tc*HMGB present a longer generation time (almost double) and the period spent on cytokinesis is five times higher in relation to control cells. The control of cytokinesis initiation in trypanosomatids is predominantly made by CIF1, Aurora B kinase, and Polo-like kinase^56–58^. In *T*. *brucei*, the disruption of CIF1 promotes an alternative cytokinesis pathway, which promotes cell division in an opposite direction of the typical cytokinesis^56^. The Aurora B kinase plays essential roles in mitosis and cytokinesis in model eukaryotes^59,60^, but in *T*. *brucei* the mutation in some key amino acids and its overexpression caused problems predominantly in mitosis^61^. Curiously, the Polo-like kinase in *T*. *brucei* was shown to be essential for kDNA segregation and also for cytokinesis^62^, two characteristics that have been impaired in our study after the induction of *Tc*HMGB (Figs 4 and 5). Whether the *Tc*HMGB overexpression disrupts the Polo-like kinase impairing cytokinesis and kDNA segregation in *T*. *cruzi* is an opened question that requires further investigation.

Finally, we also found that *Tc*HMGB is important for proper functions during *T*. *cruzi* life cycle. *Tc*HMGB overexpressing trypomastigotes infected Vero cells less efficiently than the control. Also, amastigotes duplication rates and the number of trypomastigotes released after infected-cells lysis were diminished. The whole performance of the parasite regarding host cell-infection seems to be negatively affected after *Tc*HMGB overexpression. The reduced number of released trypomastigotes can be a consequence of impaired trypomastigote infectivity, lowered amastigote replication rate and/or the capacity of the parasite to go through its life cycle forms, that is, to transform from the infective trypomastigote to the replicative amastigote and back again to trypomastigote to be finally released upon cell lysis. It cannot be easily determined if all of these processes are responsible for the final number of trypomastigotes released or to what extent, but undoubtedly, *Tc*HMGB overexpressing parasites are less efficient than the wild type to infect cells *in vitro*. Finally, metacyclogenesis, that is the transformation of epimastigotes to the infective metacyclic trypomastigotes, which occurs in the insect vector, was also reduced by the overexpression of *Tc*HMGB, as measured by an *in vitro* artificial model.

In conclusion, the results presented here suggest that *Tc*HMGB protein levels in the nucleus should be regulated for proper function of the *T*. *cruzi* cellular processes and let us propose a role for *Tc*HMGB in DNA replication and cell cycle progression control.

## Methods

### Molecular cloning of *Tc*HMGB(HA)_2_

*Tc*HMGB gene (TCDM_04259) was amplified by PCR from *T*. *cruzi* Dm28*c* genomic DNA using the following oligonucleotides: *Tc*HMGB-Fw (5′-AAGGATCCATGTCCACTGAACTAAAGTCAG-3′), *Tc*HMGB-HA-Rv (5′-AACTCGAGTTAAGCGTAATCTGGAACATCGTATGGGTAAGCGTAATCTGGAACATCGTATGGGTAGCTCGCCCTTGCAG-3′), designed to add two hemagglutinin (HA) tags at the C-terminus of the protein. Truncated protein was constructed using the following oligonucleotides: ΔN-*Tc*HMGB(HA)_2_-Fw (5′-AAGGATCCACCCAAAGGCGGCGCTCTCGCC-3′), ΔN-*Tc*HMGB(HA)_2_-Rv (5′-AACTCGAGTTAAGCGTAATCTGGAACATCGTATGGGTAAGCGTAATCTGGAACATCGTATGGGTAGCTCGCCCTTGCAG-3′). The PCR products were first cloned into pCR2.1-TOPO vector (Invitrogen) and sequenced. The coding sequences were sub cloned into pENTR-3C vector (Invitrogen) using *Bam*HI/*Xho*I restriction sites included in the oligonucleotides (underlined) and then transferred to the p*Tc*INDEX-GW vector by recombination using LR clonase II enzyme mix (Invitrogen).

### Real time PCR (qRT-PCR)

For qRT-PCR primers were designed to amplify a 58 bp fragment of *Tc*HMGB (*Tc*HMGB-Fw 5′-CGAGGTACCGCATGGAGTTC-3′ and *Tc*HMGB-Rv 5′-CTTCGTAATGATGCCCTCTATGG-3′) and glyceraldehyde 3-phosphate dehydrogenase (GAPDH) (5′-TGGAGCTGCGGTTGTCATT-3′ and 5′-AGCGCGCGTC TAAGACTTACA-3′) as an endogenous control. TRIzol reagent (Invitrogen) was used to extract total RNA from epimastigotes (1 × 10^8^ cells) and then RNA was treated with RQ1 RNase-free DNase I (Promega, Madison, WI, USA). First-strand cDNA was synthesized using the First Strand cDNA Synthesis kit (Life Technologies) according to the manufacturer’s instructions. The reactions were performed with 200 nM forward and reverse *Tc*HMGB primers or 200 nM forward and reverse GAPDH primers, SYBR Green Master Mix (Applied Biosystems, Life Technologies, Argentina) and epimastigote cDNA in triplicates. An ABI PRISM 7000 (Applied Biosystems) thermocycler was used following standard cycling conditions. The data were analyzed by the 2^−ΔΔCT^ method normalizing with GAPDH using the 7000 SDS software (Applied Biosystems).

### Polyclonal antibodies

Rabbit polyclonal antibodies against *Tc*HMGB were affinity-purified from antisera obtained as described in our previous work^16^.

### Parasite cultures

*T*. *cruzi* Dm28c epimastigotes were cultured at 28 °C in LIT medium (5 g/L liver infusion, 5 g/L bacto-tryptose, 68 mM NaCl, 5.3 mM KCl, 22 mM Na_2_HPO_4_, 0.2% (w/v) glucose and 0.002% (w/v) hemin) supplemented with 10% (v/v) heat-inactivated, UV-irradiated Fetal Calf Serum (FCS) (Internegocios S.A, Argentina).

### Transgenic parasites generation

Epimastigotes from *T*. *cruzi* Dm28*c* were transfected with the pLEW13 plasmid to generate parasites expressing T7 RNA polymerase and the Tet repressor using a standard electroporation method^21^. Briefly, epimastigotes were cultured in LIT medium at 28 °C to a final concentration of 3–5 × 10^7^ parasites/ml. Then, parasites were harvested by centrifugation at 1500 g for 5 min at room temperature, washed twice with phosphate buffered saline (PBS) and resuspended in 0.35 ml transfection buffer (0.5 mM MgCl_2_, 0.1 mM CaCl_2_ in PBS, pH 7.5) to a density of 1 × 10^8^ cells/ml for each transfection. Electroporation was performed in a 0.2 cm gap cuvette (Bio-Rad) with ~40 μg of plasmid DNA added to a final volume of 400 μl. The parasite-DNA mixture was kept on ice for 25 min and subjected to a 450 V, 500 μF pulse using GenePulser II (Bio-Rad, Hercules, USA). After electroporation, cells were transferred into 3 ml of LIT medium containing 10% FCS, maintained at room temperature for 15 minutes and then incubated at 28 °C. Geneticin (G418; Life Technologies) was added at a concentration of 200 μg/ml, and parasites were incubated at 28 °C. After selection, pLEW13 transfected epimastigotes were maintained in the presence of 200 μg/ml of G418. This parental cell line was then transfected with p*Tc*INDEX-GW-*Tc*HMGB construct following a similar protocol and transgenic parasites were obtained after 3 weeks of selection with 100 μg/ml G418 and 200 μg/ml Hygromycin B (Sigma).

### *In vitro* metacyclogenesis

Metacyclic trypomastigotes were obtained *in vitro* using chemically defined conditions as described previously^63^. Briefly, exponential epimastigotes were washed with PBS and resuspended in TAU medium (190 mM NaCl, 17 mM KCl, 2 mM MgCl_2_, 2 mM CaCl_2_, 8 mM phosphate buffer pH 6.0) to a density of 5 × 10^8^ parasites/ml with and without Tet (0.5 μg/ml) for 2 hs at 28 °C. Then, they were diluted 1:100 in TAU3AAG Medium (TAU medium plus 10 mM Glucose, 2 mM L-Aspartic Acid, 50 mM L-Glutamic Acid and 10 mM L-Proline) and incubated at 28 °C for 72 hours in the absence or presence of Tet. Parasites were collected, fixed in 4% paraformaldehyde solution and stained with Giemsa to be visualized with a Nikon Eclipse Ni-U microscope and counted using ImageJ software^64^. Only parasites with a fully elongated nucleus and rounded kinetoplast at the posterior end of the parasite were considered as metacyclic forms. Five hundred parasites from each triplicate were counted and the experiment was repeated three times.

### *T*. *cruzi* infection of Vero cells

Vero cells (ATCC CCL-81) were cultured in Dulbecco’s Modified Eagle Medium (DMEM) (ThermoFisher), supplemented with 2 mM L-glutamine, 10% FCS, 100 U/ml penicillin and 100 μg/ml streptomycin. For the first round of infection, metacyclic trypomastigotes were obtained by spontaneous differentiation from late-stationary phase cultured epimastigotes at 28 °C. Cell-derived trypomastigotes were obtained by infection with metacyclic trypomastigotes in Vero cell monolayers. For the infection and amastigote proliferation experiments, we used cell-derived trypomastigotes released from the second round of infection. Trypomastigotes were collected from the supernatant of the infected cells culture, harvested by centrifugation at 5000 × g for 10 min at room temperature, resuspended in DMEM and counted in a Neubauer chamber. When indicated, the parasites were pre-incubated with Tet (0.5 μg/ml) for 2 hs in order to allow protein induction and a new monolayer of cultured Vero cells was infected with a MOI of 10:1. After a 6h-incubation at 37 °C, the free trypomastigotes were removed by washes with PBS. This pre-treatment of trypomastigotes with Tetracycline prior to infection and during the 6 h-incubation to allow invasion is indicated with a “+” before the bar “(+/)”. Then, infected Vero cell cultures were incubated in DMEM supplemented with 2% FCS with or without Tet (0.5 μg/ml) for two days. The presence of Tet during this incubation period when amastigotes replicate inside the infected cell is indicated by the “+” after the bar “(/+)”. The experiments were stopped by cell fixation with methanol and the percentage of infected cells and the mean number of amastigotes per infected cell was determined by direct slide counting (Nikon Eclipse Ni-U microscope). Giemsa staining was used for amastigotes visualization and approximately 800 cells were counted per slide.

### Western blot

Protein extracts were fractioned in SDS-PAGE and transferred to a nitrocellulose membrane. Transferred proteins were visualized with Ponceau S staining. Membranes were treated with 10% non-fat milk in PBS for 2 hours and then incubated with specific antibodies diluted in 0.5% Tween20 in PBS (PBS-T) for 3 hours. Antibodies used were: rat monoclonal anti-HA (ROCHE), affinity-purified rabbit polyclonal anti-*Tc*HMGB, mouse monoclonal anti-trypanosome α-tubulin clone TAT-1 (a gift from K. Gull, University of Oxford, UK). Bound antibodies were detected using peroxidase-labeled anti-mouse, anti-rabbit IgG (GE Healthcare), anti-mouse IgG (GE Healthcare) or anti-rat IgG (Thermo Scientific) and developed using ECL Prime kit (GE Healthcare) according to manufacturer’s protocols. Immunoreactive bands were visualized by autoradiography, photographed and images were processed with Adobe Photoshop 6.0.

### Immunofluorescence

Mid-log epimastigotes were harvested by centrifugation at 1500 xg for 5 min at room temperature and washed twice prior to fixation in 4% paraformaldehyde solution. Fixed parasites were placed on a coverslip pre-coated with poly L-lysine for 20 min and then washed with PBS. Permeabilization was done with 0.2% Triton X-100 solution for 10 min. After washing with PBS, parasites were incubated with the appropriate primary antibody diluted in 1% BSA in PBS for 2 hs at room temperature. Non-bound antibodies were washed with PBS-T and then the slides were incubated with anti-rat IgG::FITC (Life Technologies) and anti-rabbit IgG::Cy3 (Life Technologies) conjugated antibodies and 2 μg/ml 4′,6-diamidino-2-fenilindol (DAPI) for 1 hour. The slides were washed again with PBS-T and mounted with VectaShield (Vector Laboratories). Images were acquired with a confocal microscope ZEISS LSM 880. Adobe Photoshop CS and ImageJ software were used to process all images.

### Ultrastructural analysis

#### Transmission electron microscopy (TEM)

Parasites were washed twice in PBS and fixed in 2.5% (w/v) glutaraldehyde in 0.1 M cacodylate buffer (pH 7.2) for 1 h. Then, cells were washed in 0.1 M cacodylate buffer (pH 7.2) and post fixed for 1 h in 1% (w/v) osmium tetroxide, 0.8% potassium ferrocyanide and 5 mM CaCl2 in 0.1 M cacodylate buffer. After post fixation, cells were washed in the same buffer, dehydrated in a series of increasing acetone concentrations and embedded in Epon, first as a mixture of Epon and acetone (1:1) and then as pure Epon. Ultrathin sections were obtained using an Ultracut Reichert Ultramicrotome and mounted on 400-mesh copper grids. Samples were stained with uranyl acetate and lead citrate and then analyzed using a Zeiss 900 transmission electron microscope. Measurements of the total nucleus area, as well as nucleolar domains, such as nucleolus, heterochromatin and euchromatin, were made in images obtained by transmission electron microscopy using ImageJ software. First we delimited the nucleus, then the nucleolus and the heterochromatin region. The euchromatin region corresponds to the total nuclear area minus those occupied by the nucleolus and heterochromatin regions. Statistics were calculated using the unpaired t test with Welch’s correction in GraphPad Prism 6 software (GraphPad Software). P values less than 0.05 were considered statistically significant.

#### Scanning electron microscopy (SEM)

Parasite processing was carried out using glass coverslips pre-coated with 1 mg/ml poly-L-lysine. Cells were fixed for 1 h in 2.5% glutaraldehyde diluted in cacodylate buffer [0.1 M (pH 7.2)] and then were adhered to coverslips, post-fixed for 1 h with 1% osmium tetroxide diluted in cacodylate buffer. Samples were dehydrated in a graded alcohol series (50%, 70%, 90%, and two exchanges of 100% ethanol for 10 min each step) and then were critical-point dried in a Leica EM CPD030 apparatus (Leica, Wetzlar, Germany). Specimens were coated with platinum in a Leica EM SCD050 before visualization using a Zeiss EVO 40 VP scanning electron microscope. Measurements of cells lengths were made using the program AxioVision4 and were based upon the SEM images. Statistics were calculated using the Wilcoxon-Mann-Whitney test in GraphPad Prism 6 software (GraphPad Software). P values less than 0.05 were considered statistically significant.

### Micrococcal nuclease assay

Micrococcal nuclease (MNase) assay was performed as described previously^37^ with slight modifications. Briefly, 10^8^ parasites were washed in lysis buffer (1 mM potassium L-glutamate, 250 mM Sucrose, 2.5 mM CaCl_2_, 1 mM PMSF) and resuspended in the same buffer containing 0.1% Triton X-100. The supernatant was discarded and the nuclei-containing-pellet washed twice in lysis buffer without detergent. Samples were incubated with 1U of MNase (Thermo Scientific) for 0, 5, 25 minutes at 37 °C, and the reaction was stopped with 2 mM EDTA-EGTA. Then, 10 μl of proteinase K (20 mg/ml) was added and incubated at 56 °C for 3 hs. Subsequently, DNA was purified by ethanol precipitation after phenol/chloroform extraction and analyzed in a 1.5% agarose gel stained with ethidium bromide, visualized under UV light and photographed with a Nikon 3200 digital camera. Images were processed with Adobe Photoshop 6.0.

### Analysis of the cell cycle

Cell cycle progression of parasites was analyzed by flow cytometry. One million cells were fixed with cold 70% ethanol and then washed with PBS and stained with 20 μg/ml Propidium Iodide (PI) in buffer K (0.1% sodium citrate, 0.02 mg/ml RNAse A (Sigma), and 0.3% NP-40. Ten thousand events per sample were acquired using BD Cell Sorter BD FACSAria II. Results were analyzed using WinMDi, Cylchred and FlowJo software.

Formaldehyde-fixed and DAPI-stained exponentially growing epimastigotes forms of *T*. *cruzi* Dm28c/p*Tc*INDEX-GW-HMGB(HA)_2_ [induced with tetracycline (Tet+), and non-induced (Tet -)] were examined under an Olympus BX51 fluorescent microscope (Olympus, Japan) (100x oil objective) to observe the profile of organelles that contain DNA (nucleus and kinetoplast). To estimate the duration of mitosis (M) and cytokinesis (C), we used the Williams (1971) equation:

$$x=\frac{\mathrm{ln}(1-y/2)}{-\alpha },$$

where x is the cumulative time within the cycle until the end of the stage in question, y is the cumulative % of cells up to and including the stage in question (expressed as a fraction of one unit), and α is the specific growth rate.

To estimate the G2 + M phases, we used an EdU pulse (1 h) and then collected parasites every 15 min until a parasite containing two EdU-labeled nuclei (2N2K or 2N1K) were observed. The difference between this value and the duration of mitosis previously calculated corresponds to G2 phase duration. The duration of S phase was estimated according to the Stanners and Till (1960) equation:

$$S=\frac{1}{\alpha }\,\mathrm{ln}[L+{e}^{\alpha (Z)}]-(Z+t),$$

where *L* is the proportion of cells exhibiting EdU-labeled nuclei, α = ln 2/T (*T* = doubling time expressed in hours), Z = G2 + M + cytokinesis, and *t* is the duration of the EdU labeling period in hours. Finally, the duration of G1 phase was estimated by the difference between the doubling time and the sum of the remaining phases.

### Statistical analysis

Statistical analysis of the data was carried out using GraphPad Prism version 6.0.

## Electronic supplementary material

Supplementary information

## Data Availability

No datasets were generated or analyzed during the current study.
