# Supplementary material for: Overexpression of Trypanosoma cruzi High Mobility Group B protein (TcHMGB) alters the nuclear structure, impairs cytokinesis and reduces the parasite infectivity
Source: Sci Rep. 2019 Jan 17;9:192. doi: 10.1038/s41598-018-36718-0 (PMC6336821; doi:10.1038/s41598-018-36718-0)

Supplementary information

Overexpression of *Trypanosoma cruzi* High Mobility Group B protein (*Tc*HMGB) alters the nuclear structure, impairs cytokinesis and reduces the parasite infectivity

Luis Emilio Tavernelli[1], Maria Cristina M. Motta[3], Camila Silva Gonçalves[3], Marcelo Santos da Silva[4,5]; Maria Carolina Elias[4,5], Victoria Lucia Alonso[2], Esteban Serra[1,2], Pamela Cribb*[1,2]

Figure S1: Inducible expression of *Tc*HMGB(HA)$_2$. (A) Equal amounts of total lysate from *T. cruzi* Dm28c/p*Tc*INDEX-GW-*Tc*HMGB(HA)$_2$ epimastigotes incubated for 12h in the absence (Tet-) or presence (Tet+) of 0.5 µg/ml of tetracycline were loaded on SDS/PAGE and then transferred to nitrocellulose for western blot analysis. Rat anti-HA monoclonal antibodies (a-HA), purified polyclonal rabbit anti-*Tc*HMGB (a-*Tc*HMGB) and mouse anti-tubulin (a-Tubulin) were used as indicated; "*" corresponds to the HA-tagged-*Tc*HMGB and "+" to the endogenous *Tc*HMGB. The intensity of the *Tc*HMGB bands was quantified and normalized to the corresponding tubulin intensity. The bar graph bellow represents the relative intensity of the bands. Full-length blots are presented in Supplementary Fig. S4 (B) Quantitation of *Tc*HMGB transcript. Total RNA was purified from induced (Tet+) or non-induced (Tet-) Dm28c/p*Tc*INDEX-GW-*Tc*HMGB(HA)$_2$ epimastigotes, and relative *Tc*HMGB mRNA levels were determined by qRT-PCR using GAPDH as housekeeping gene with the ΔΔCt method. Data are expressed as the fold change relative to the control (Tet-) group (white column) and presented as Mean ± SD corresponding to three independent experiments; *p<0.05 (Student t test). Transcript levels are shown in arbitrary units. (C) Anti-HA monoclonal antibodies were used in western blot assays to show the inducible overexpression of *Tc*HMGB(HA)$_2$ in epimastigotes (E), amastigotes (A) and trypomastigotes (T). Tet-, non-induced; Tet+, induced with 0.5ug/ml for 12h. The intensity of each band was calculated with the software ImageJ and normalized to a-tubulin bands. On the right, a graphical representation of the normalized *Tc*HMGB(HA)$_2$ tagged protein levels. (D) Immunofluorescence confocal microscopy of induced (0.5 µg/ml Tet, 12h) epimastigotes, trypomastigotes and amastigotes using rat anti-HA (a-HA) with FITC-conjugated anti-rat antibodies (green), rabbit anti-*Tc*HMGB (a-*Tc*HMGB) with Cy3-conjugated anti-rabbit antibodies (red) and DAPI (blue). Scale bar: 1 µm. Arrows indicate the nucleolar region that is not labeled in replicative forms.

Figure S2: Control assays were performed with the Inducible expression of the mutant *Tc*HMGB(HA)$_2$ (ΔN-*Tc*HMGB(HA)$_2$). (A) Schematic representation of the tagged *Tc*HMGB and truncated mutant construction ΔN-*Tc*HMGB(HA)$_2$ and western blot with antibodies directed to the HA tag and specific anti-*Tc*HMGB. Overexpression of ΔN-*Tc*HMGB(HA)$_2$ was induced by Tet (0.5 µg/ml, 12h) as can be seen in the western blot. (B) The growth curve of induced vs. non-induced epimastigotes showed a slight difference, in contrast to the high decrease observed after induction of the full-length *Tc*HMGB overexpression (compare to Fig3). Values represent mean ± SD of 3 independent replicates, *p<0.05, ***p< 0.0005, ****p<0.00005 (Student t test). (C) Analysis of chromatin isolated from ΔN-*Tc*HMGB(HA)$_2$ epimastigotes non-induced (-, lanes 1,3,5) or induced (+, lanes 2,4,6) with tetracycline (Tet). Chromatin from an equal number of cells was isolated and digested with 1 unit of micrococcal nuclease (MNase) for 0 (lanes 1,2), 5 (lanes 3,4) and 25 minutes (lanes 5,6). Equal amounts of DNA were loaded on an ethidium bromide-stained 1% agarose gel. A representative experiment is shown, where the characteristic ladder pattern is observed and digestion products corresponding to DNA that has been bound to mono- di- and tri-nucleosomes are indicated. No difference was observed between induced and non-induced parasites. (D) The deletion of the N-term domain containing the NLS directs the heterologous protein to the cytoplasm, excluded from the nucleus and so unable to interact with nuclear chromatin. (E) The overexpression of the mutant protein ΔN-*Tc*HMGB(HA)$_2$ does not affect significantly the cell cycle progression. (F) *In vitro* metacyclogenesis was performed using TAU medium of the ΔN-*Tc*HMGB(HA)$_2$ parasites non-induced (-) or induced (+) with 0.5µg/ml Tet for 96 h. Results are expressed as mean ± SD of triplicates; ***p < 0.005 (Student t test). (G-I). Infectivity on Vero cells, amastigote replication and trypomastigotes released after infection were not affected by the mutant *Tc*HMGB overexpression. Results are expressed as mean ± SD of triplicates. Statistical analysis of the data was carried out using one-way ANOVA, *p < 0.05, **p < 0.001 and ***p < 0.005.

Fig S3: Tetracycline and GFP overexpression does not affect the cell cycle progression. (A) On the left, growth curve of epimastigotes Dm28c WT with (red square) and without (green circle) Tet. On the right, growth curve of epimastigotes transfected with p*Tc*INDEX-GW-GFP with and without Tet (B) Flow Cytometry analysis of cultured epimastigotes Dm28c WT and Dm28c/p*Tc*INDEX-GW-GFP cultured with 0.5ug/ml tetracycline at different times. Graphs represent number of events vs. propidium iodide absorbance (PI-A). Red (Tet+), green (Tet-).

Fig S4: Uncropped gels and western blots from previous figures.

All Statistical analysis were performed with GraphPad Prism 6 software.

Fig S1

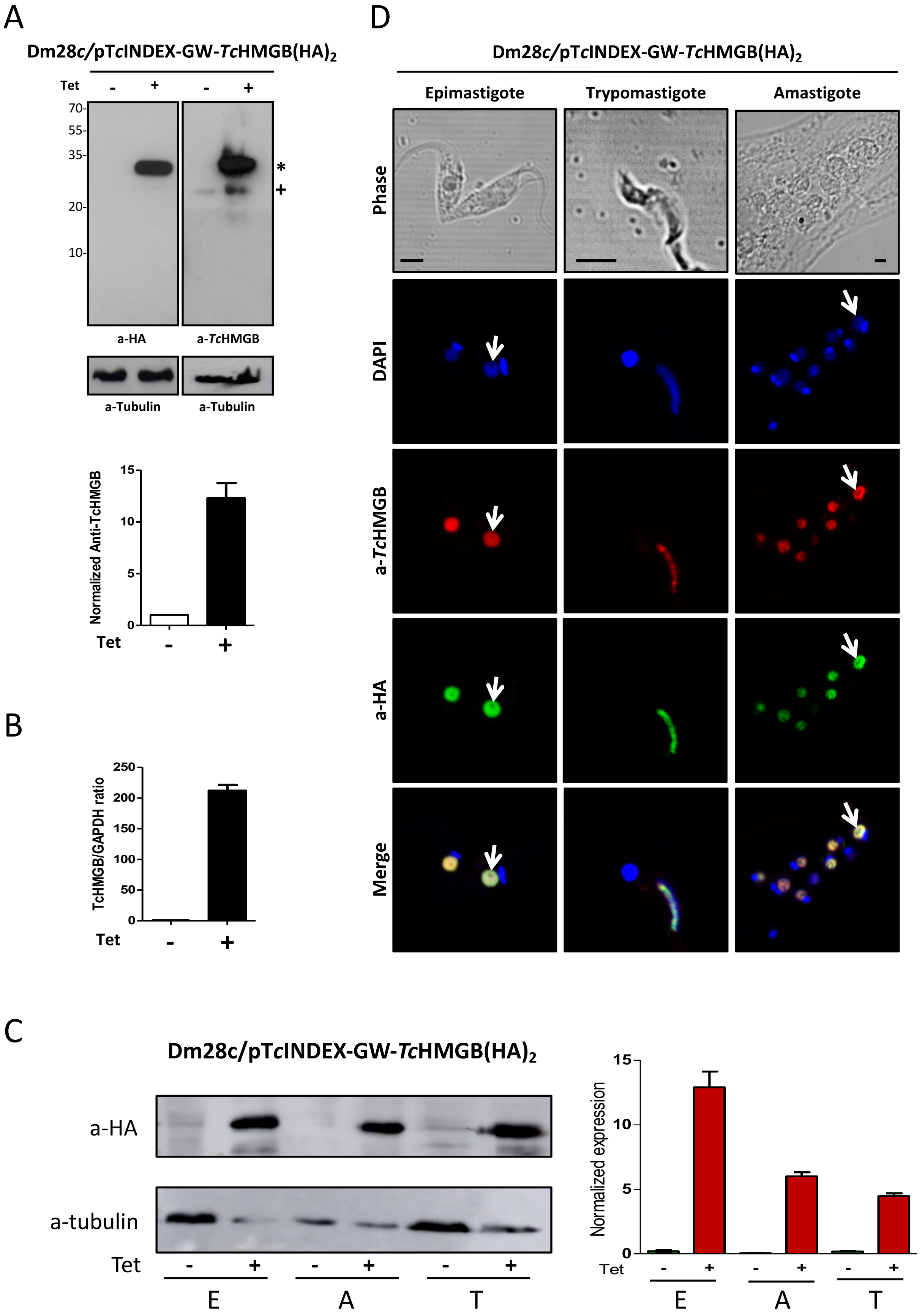
A
Dm28c/pTcINDEX-GW-TcHMGB(HA)2
Tet - + - +
70-
55-
35-
20-
10-
*
+
a-HA
a-TcHMGB
a-Tubulin
a-Tubulin
Normalized Anti-TcHMGB
15
10
5
0
Tet - +
B
TcHMGB/GAPDH ratio
250
200
150
100
50
0
Tet - +
C
Dm28c/pTcINDEX-GW-TcHMGB(HA)2
a-HA
a-tubulin
Tet - + - + - +
E A T
Normalized expression
15
10
5
0
Tet - + - + - +
E A T
D
Dm28c/pTcINDEX-GW-TcHMGB(HA)2
Epimastigote
Trypomastigote
Amastigote
Phase
DAPI
a-TcHMGB
a-HA
Merge

Fig S2

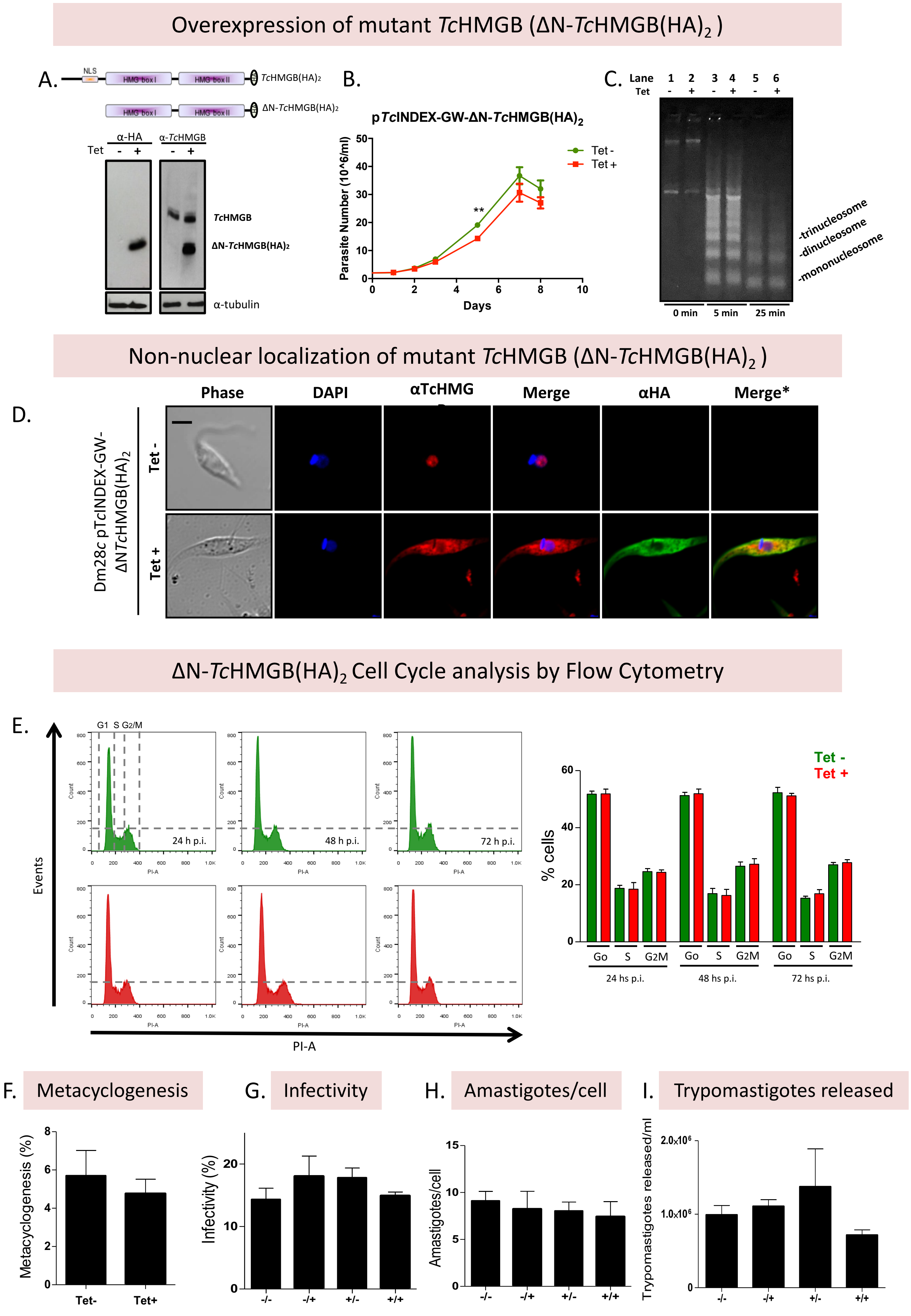

Overexpression of mutant TcHMGB (ΔN-TcHMGB(HA)2)
A.
NLS
HMG box I
HMG box II
TcHMGB(HA)2
ΔN-TcHMGB(HA)2
α-HA
α-TcHMGB
Tet - + - +
TcHMGB
ΔN-TcHMGB(HA)2
α-tubulin
B.
pTcINDEX-GW-ΔN-TcHMGB(HA)2
Parasite Number (10^6/ml)
Days
Tet -
Tet +
**
C.
Lane 1 2 3 4 5 6
Tet - + - + - +
-trinucleosome
-dinucleosome
-mononucleosome
0 min
5 min
25 min
Non-nuclear localization of mutant TcHMGB (ΔN-TcHMGB(HA)2)
Phase
DAPI
αTcHMG
Merge
αHA
Merge*
D.
Dm28c pTcINDEX-GW-ΔNTcHMGB(HA)2
Tet -
Tet +
ΔN-TcHMGB(HA)2 Cell Cycle analysis by Flow Cytometry
E.
G1 S G2/M
24 h p.i.
48 h p.i.
72 h p.i.
Events
PI-A
Count
Tet -
Tet +
% cells
Go S G2M Go S G2M Go S G2M
24 hs p.i.
48 hs p.i.
72 hs p.i.
F. Metacyclogenesis
Metacyclogenesis (%)
Tet-
Tet+
G. Infectivity
Infectivity (%)
-/- -/+ +/- +/+
H. Amastigotes/cell
Amastigotes/cell
-/- -/+ +/- +/+
I. Trypomastigotes released
Trypomastigotes released/ml
2.0x10^6
1.0x10^6
-/- -/+ +/- +/+

Fig S3

Effect of Tetracycline on DM28c (wt) and p*Tc*INDEX-GW-GFP

A Epimastigotes growth curve

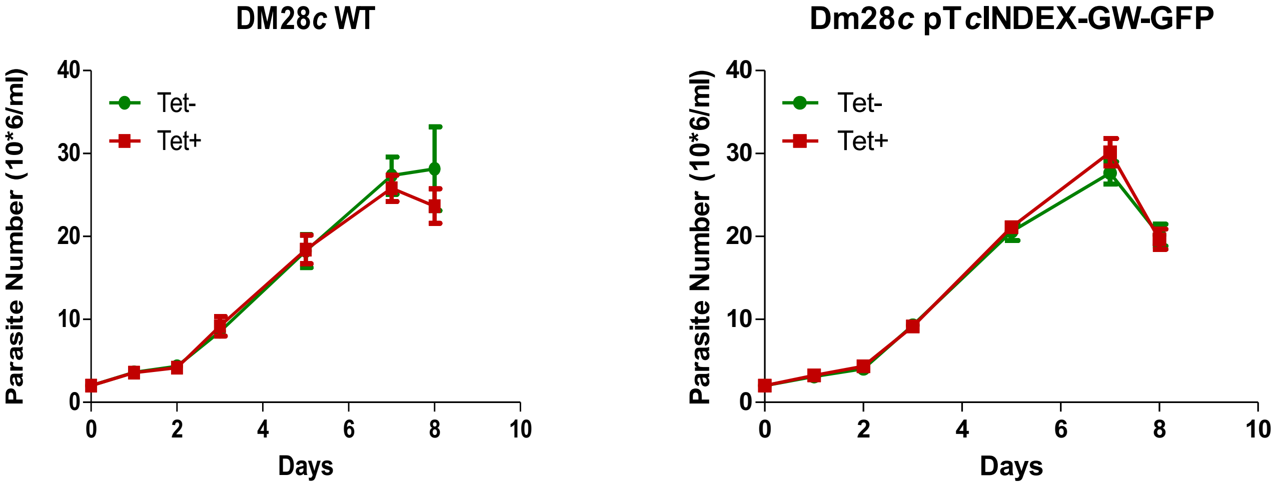

B Cell Cycle anaysis

Dm28c (wt)

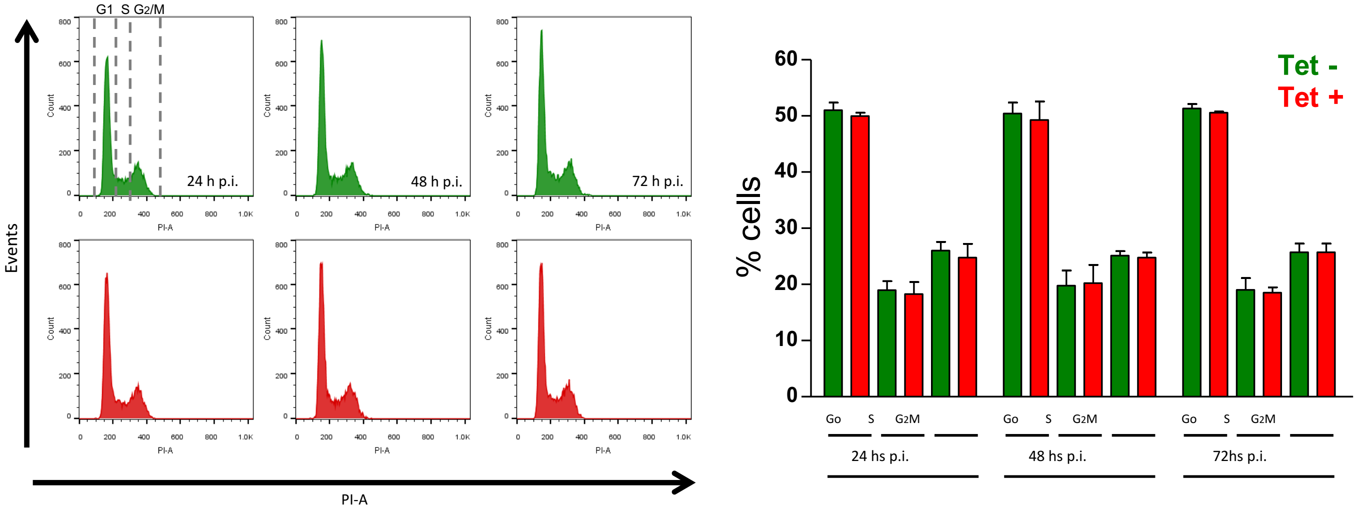

p*Tc*INDEX-GW-GFP

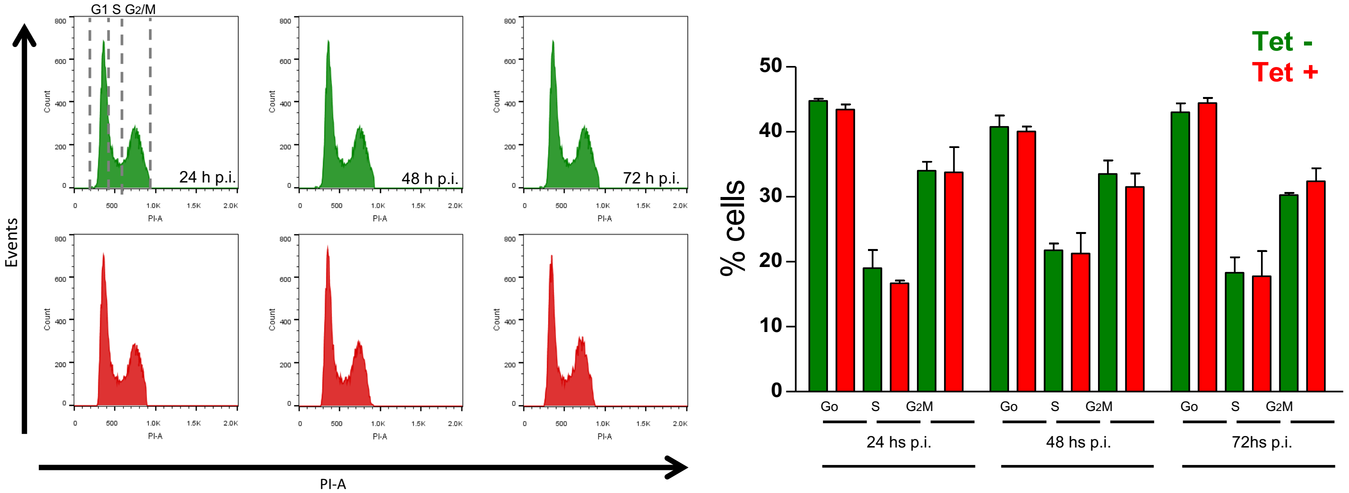

Fig S4

Uncropped gels and Westerns Blots

Uncropped Wester blots and Ponceau from Figure S1

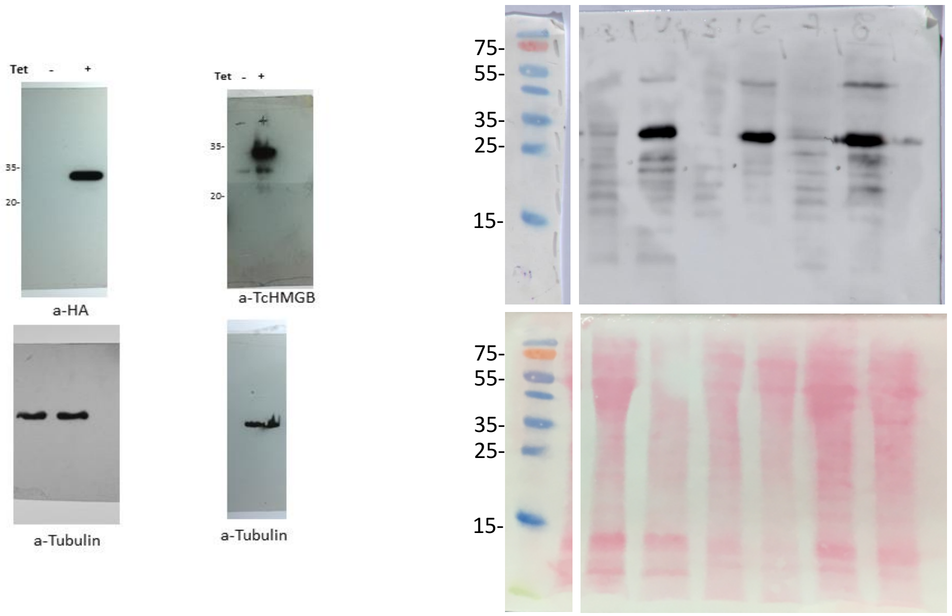

Uncropped wester blot from figure S2

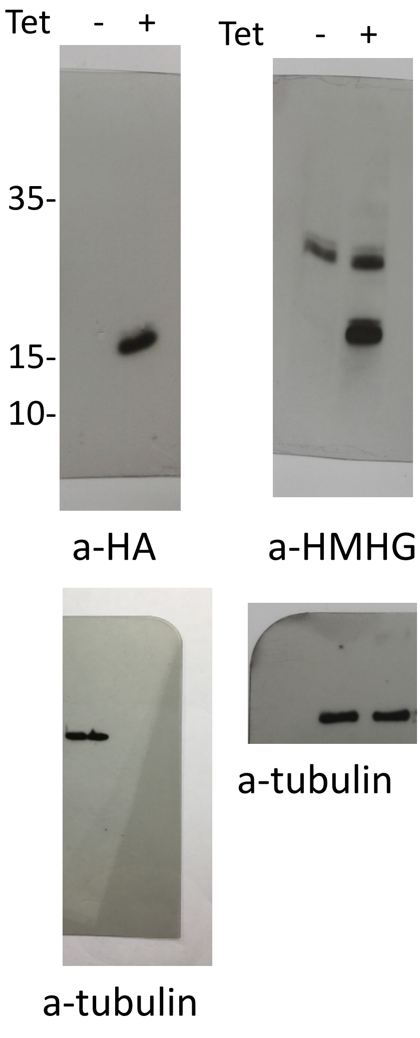

Uncropped agarose gel from Figure 2

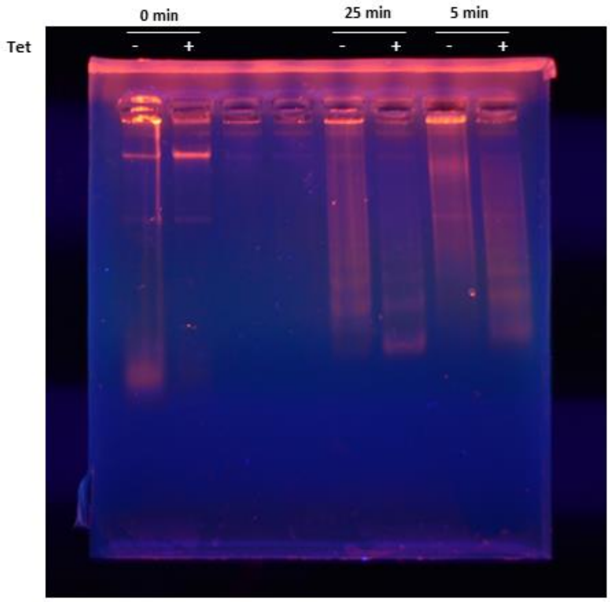

Supplement: Supplementary file 1 — Supplementary information [file 41598_2018_36718_MOESM1_ESM.pdf]
